# Human fecal and pathogen exposure pathways in rural Indian villages and the effect of increased latrine coverage

**DOI:** 10.1016/j.watres.2016.05.015

**Published:** 2016-09-01

**Authors:** Mitsunori Odagiri, Alexander Schriewer, Miles E. Daniels, Stefan Wuertz, Woutrina A. Smith, Thomas Clasen, Wolf-Peter Schmidt, Yujie Jin, Belen Torondel, Pravas R. Misra, Pinaki Panigrahi, Marion W. Jenkins

**Affiliations:** a Dept. of Civil and Environmental Engineering, University of California, Davis, One Shields Avenue, Davis, CA, USA; b Dept. of Veterinary Medicine and Epidemiology, School of Veterinary Medicine, University of California, Davis, CA, USA; c Singapore Centre for Environmental Life Sciences Engineering (SCELSE), Nanyang Technological University, 60 Nanyang Drive, Singapore; d School of Civil and Environmental Engineering, Nanyang Technological University, 50 Nanyang Avenue, Singapore; e Dept. of Environmental Health, Rollins School of Public Health, Emory University, Atlanta, GA, USA; f Asian Institute of Public Health, Bhubaneswar, Odisha, India; g Faculty of Infectious and Tropical Diseases, London School of Hygiene and Tropical Medicine, Keppel Street, London WC1E 7HT, UK; h Dept. of Epidemiology, Center for Global Health and Development, College of Public Health, University of Nebraska Medical Center, Omaha, NE, USA; i Dept. of Pediatrics, Center for Global Health and Development, College of Public Health, University of Nebraska Medical Center, Omaha, NE, USA

**Keywords:** Microbial source tracking, *Bacteroidales*, Child diarrhea prevalence, Improved sanitation, Drinking water contamination, Hand contamination

## Abstract

Efforts to eradicate open defecation and improve sanitation access are unlikely to achieve health benefits unless interventions reduce microbial exposures. This study assessed human fecal contamination and pathogen exposures in rural India, and the effect of increased sanitation coverage on contamination and exposure rates. In a cross-sectional study of 60 villages of a cluster-randomized controlled sanitation trial in Odisha, India, human and domestic animal fecal contamination was measured in community tubewells and ponds (n = 301) and via exposure pathways in homes (n = 354), using *Bacteroidales* microbial source tracking fecal markers validated in India. Community water sources were further tested for diarrheal pathogens (rotavirus, adenovirus and *Vibrio cholerae* by quantitative PCR; pathogenic *Escherichia coli* by multiplex PCR; *Cryptosporidium* and *Giardia* by immunomagnetic separation and direct fluorescent antibody microscopy). Exposure pathways in intervention and control villages were compared and relationships with child diarrhea examined. Human fecal markers were rarely detected in tubewells (2.4%, 95%CI: 0.3–4.5%) and ponds (5.6%, 95%CI: 0.8–10.3%), compared to homes (35.4%, 95%CI: 30.4–40.4%). In tubewells, *V. cholerae* was the most frequently detected pathogen (19.8%, 95%CI: 14.4–25.2%), followed by *Giardia* (14.8%, 95%CI: 10.0–19.7%). In ponds, *Giardia* was most often detected (74.5%, 95%CI: 65.7–83.3%), followed by pathogenic *E. coli* (48.1%, 95%CI: 34.8–61.5%) and rotavirus (44.4%, 95%CI: 34.2–54.7%). At village-level, prevalence of fecal pathogen detection in community drinking water sources was associated with elevated prevalence of child diarrhea within 6 weeks of testing (RR 2.13, 95%CI: 1.25–3.63) while within homes, higher levels of human and animal fecal marker detection were associated with increased risks of subsequent child diarrhea (*P* = 0.044 and 0.013, respectively). There was no evidence that the intervention, which increased functional latrine coverage and use by 27 percentage points, reduced human fecal contamination in any tested pathway, nor the prevalence of pathogens in water sources. In conclusion, the study demonstrates that (1) improved sanitation alone may be insufficient and further interventions needed in the domestic domain to reduce widespread human and animal fecal contamination observed in homes, (2) pathogens detected in tubewells indicate these sources are microbiologically unsafe for drinking and were associated with child diarrhea, (3) domestic use of ponds heavily contaminated with multiple pathogens presents an under-recognized health risk, and (4) a 27 percentage point increase in improved sanitation access at village-level did not reduce detectable human fecal and pathogen contamination in this setting.

## Highlights

• Application of *Bacteroidales* MST to evaluate improved sanitation impacts.
• Widespread human and animal fecal contamination detected in homes.
• Pathogens detected in drinking sources associated with subsequent child diarrhea.
• Public ponds used domestically were heavily contaminated with multiple pathogens.
• No decrease in human fecal or pathogen contamination from increased latrine coverage.

## 1 Introduction

Despite reductions in global child mortality, an estimated 6.6 million children under age five still died in 2012, of which 22% were in India (UNICEF, 2013). Diarrheal diseases associated with poor sanitation are a leading cause of child deaths in developing countries, accounting for an estimated 13% of the deaths in India (Liu et al., 2012). While proper excreta disposal through good sanitation is necessary to reduce this disease burden, the impact of a specific sanitation intervention on health within a given setting is not always guaranteed as demonstrated by two recent evaluations of the Indian government's rural sanitation program (Clasen et al., 2014, Patil et al., 2014). Lack of measurable health impact may occur when the sanitation intervention does not adequately disrupt fecal-oral pathogen transmission pathways and/or address critical sources operating in the intervention setting. Pathways and sources have generally been difficult to assess using traditional fecal indicator bacteria (FIB) of microbial exposure such as total coliforms, thermotolerant coliforms, *Escherichia coli*, and members of the genus *Enterococcus* (the enterococci). FIB can originate from both humans and animals, and have been found in environments where fecal contamination is improbable, due to the presence of naturalized FIB and the ability to grow outside their hosts (Ishii et al., 2006, Leclerc et al., 2001, Power and Nagy, 1999). To move understanding forward, efforts using new research tools are needed to shed light on gastro-intestinal pathogen transmission pathways, pathogen sources, and the mechanisms by which different water, sanitation and hygiene interventions interrupt key pathways in diarrheal disease burden settings.

In this research, we apply advanced microbial source tracking (MST) techniques to assess human-associated fecal exposure in the public and domestic domains of 30 intervention and 30 control villages of a large cluster-randomized controlled trial of impacts of improved household sanitation on health in rural India (the Sanitation Trial) (Clasen et al., 2012), and examine the effect of increased intervention latrine coverage on fecal exposure via different pathways in the community. We employ a set of MST host-associated fecal *Bacteroidales* assays recently validated to distinguish and quantify human versus non-human livestock and other domestic animal sources of fecal contamination in the study area (Odagiri et al., 2015).

The primary objectives were to (1) measure prevalence of human and animal fecal contamination of community water sources, and of household stored drinking water and mothers' and children's hands; (2) assess the microbiological safety of community water sources by measuring locally important child diarrhea-causing pathogens; and (3) examine the effect of increased latrine coverage in intervention villages on contamination and pathogen exposure rates. In a secondary analysis, we explore associations between detected human and animal fecal contamination in homes and household child diarrhea, and between observed pathogen contamination of village water sources and village-wide child diarrhea prevalence to shed light on potential diarrheal pathogen transmission pathways and sources operating in study communities.

## 2 Materials and methods

### 2.1 Study setting

The study was carried out in Puri District in Odisha, India in a subset of 30 intervention and 30 control villages of the Sanitation Trial. The Sanitation Trial design, pour-flush latrine construction intervention, study setting and population characteristics have been described elsewhere (Boisson et al., 2014, Clasen et al., 2012). Briefly, key characteristics of the study population include (1) low sanitation coverage prior to the intervention (10% of households); (2) high access to improved drinking water sources such as public (deep groundwater) and private (shallow groundwater) tubewells (82% of households); (3) daily usage of open ponds for non-drinking purposes including anal cleansing after defecation, bathing, brushing teeth and domestic hygiene activities (>50% of households); and (4) livestock ownership (59% of households), comprising cattle (most frequently owned), sheep, goat and buffalo.

The latrine promotion and construction program in intervention villages was undertaken between January 2011 and January 2012, yielding mean village-level coverage with functional latrines in the intervention and control villages of the present study of 37.5% and 10.2%, respectively.

### 2.2 Village and household selection

Each Sanitation Trial intervention village (n = 50) was uniquely paired with one nearby control village (n = 50), and 30 pairs randomly selected into this study. Testing occurred in each village of a pair on consecutive days to minimize spatial and temporal confounding over the monsoon season study period. Twelve pairs were sampled in 2012 from June 19th to July 26th; 18 pairs in 2013 from June 26th to August 22nd. In each village, households with a child under 5 enrolled in the Sanitation Trial health surveillance study were stratified based on drinking public or private tubewell water, and three households in each stratum randomly selected for testing. In total, 354 households were sampled.

### 2.3 Community water source selection

Two public tubewells (deep groundwater), two private tubewells (shallow groundwater) and two open ponds (surface water) in each village were tested, unless fewer existed. Further details are provided in Supplemental Material. Sources were each sampled once, during or just after household sampling between 8 and 11 a.m. In total, 111 public and 98 private tubewells, and 94 open ponds were sampled.

### 2.4 Sample collection

#### 2.4.1 Community water sources

A 20-L sample was collected for molecular analysis as previously described (Schriewer et al., 2015). For thermotolerant coliform (TTC; also known as fecal coliform) measurement, an additional 100-mL sample was collected in a sterile 4-oz Whirl-Pak (NASCO Corp., Fort Atkinson, WI). Samples were placed on ice, transported to a laboratory in Bhubaneshwar, and processed within 8 h of collection.

#### 2.4.2 Household stored drinking water and hand rinses

Approximately 500 mL of stored drinking water (SDW) was collected for molecular analysis and a further 100 mL separately collected for TTC measurement using sterile 69-oz and 4-oz Whirl-Paks, respectively. The collectable amount of SDW was limited by the small volumes of stored drinking water and unwillingness of households to give all of it for research purposes. Hand rinses (HR) were obtained from the mother and youngest child following a published protocol (Pickering et al., 2010). Because recent household activities can significantly affect the level of hand fecal contamination (Devamani et al., 2014, Pickering et al., 2011), we standardized HR collection times, resulting in 76% and 75%, respectively, of intervention and control villages’ HR samples collected when mothers were preparing or eating foods.

### 2.5 Sample processing

#### 2.5.1 Community water source samples

Each 20-L sample was filtered on the day of collection using a hollow fiber ultrafiltration system according to a published protocol (Bambic et al., 2011). For molecular analysis, 2.5 mL of the RNA/DNA stabilizing agent RNALater (Qiagen, Valencia, CA) was added to the same amount of the filtration retentate, mixed well, and stored at −70 °C for up to two months until transport back to the University of California, Davis (UCD) for analysis. For protozoa analysis, 50 mL of the filtration retentate was separately kept at 4 °C for up to one week. For quality assurance, 10% of samples were assigned to field blanks, filtration blanks and duplicates, and were similarly processed.

#### 2.5.2 Household stored drinking water and hand rinse samples

Samples were filtered with 47-mm, 0.45-μm Millipore HA filters (Fisher Scientific, Pittsburgh, PA, USA) for molecular analysis. Filtered volumes varied depending on the sample type and turbidity (details in Supplemental Material). Prior to molecular filtration of HR samples, an aliquot (up to 10 mL) was hygienically removed for TTC testing. After filtration, each membrane was placed into a 5-mL cryogenic tube containing 0.5 mL of RNALater. Tubes were stored at −70 °C for up to two months until transport back to UCD. Field blanks and lab filtration blanks each made up 8% of total samples.

### 2.6 Microbiological and physicochemical measurements

#### 2.6.1 Thermotolerant coliforms

All samples were analyzed for TTC within 6 h of collection using the membrane filtration method (Eaton et al., 2012). See Supplemental Material for testing details, detection limits, assigned values for non-detect (ND) and too numerous to count (TNTC) samples, and correlation with MST markers.

#### 2.6.2 Molecular analyses

DNA and RNA extraction methods, and detection methods and marker targets for each molecular assay are described in Supplemental Material. Nucleic acid extracts from all sample types were tested for total-, human- and animal-associated *Bacteroidales* using the quantitative PCR (qPCR) assays BacUni, BacHum and BacCow, respectively, following developer's protocols (Kildare et al., 2007). These assays were recently evaluated in Odisha, India, to be the most suited for distinguishing human from non-human animal (domestic and livestock) hosts in the study region (Odagiri et al., 2015). Due to observed cross-reactivity of the human-associated assay, BacHum, with dog feces (Odagiri et al., 2015), we tested all BacHum-positive samples for dog-associated *Bacteroidales* using BacCan (Kildare et al., 2007), also validated in India by Odagiri et al., to confirm absence of dog fecal contamination. BacHum was also reported by Odagiri to cross-react with chicken feces. However, only 7 out of 354 sampled households owned chickens, reducing the likelihood of false-positives from chicken feces. Results are given in gene copies (gc) per mL for water samples or per two hands for HRs. Sample limit of detection (SLOD) values for each assay are reported in Supplemental Material. Different SLOD for each sample can bias presence/absence results. For fair comparison of detection rates, therefore, we removed SLOD outlier samples (>3σ + the mean SLOD) for each sample type prior to data analysis and any sample with a concentration below the maximum SLOD of non-detected samples was classified as below the presence/absence detection limit.

#### 2.6.3 Pathogen analyses

Nucleic acid extracts from community water source samples were analyzed for rotavirus, adenovirus 40/41 and *Vibrio cholerae* possessing the *ctxA* gene via qPCR following original developers’ protocols (Blackstone et al., 2007, Jothikumar et al., 2009, Rajal et al., 2007). SLODs for each assay are provided in Supplemental Material. For protozoa, *Cryptosporidium* oocysts and *Giardia* cysts were further concentrated via immunomagnetic separation (IMS) following ultrafiltration, and enumerated using direct fluorescent antibody (DFA) microscopy as described elsewhere (Daniels et al., 2015). Community water source samples collected in 2013 and positive for TTC were analyzed for pathogenic *Escherichia coli* by three multiplex conventional PCR assays according to Mattioli et al. (2013). Seven virulent genes were detected: *aggR* (EAEC), *eaeA* (EHEC/EPEC), *stx1* (EHEC), *stx2* (EHEC), *ipaH* (EIEC and *Shigella* spp.), *lt1* (ETEC) and *st1b* (ETEC). All pathogen assays were validated on site against clinically positive stool samples or pathogen-positive isolates obtained from local diarrhea patients in the study region prior to use, and later, a subset of pathogen-positive water samples were sequenced to confirm qPCR target genes (see Supplemental Material).

#### 2.6.4 pH and turbidity

pH and turbidity of HR samples collected in 2012 and all samples collected in 2013 were measured using pH and turbidity meters (135, SYSTRONICS, India). See Supplemental Material for descriptive, correlation, and comparison statistics.

### 2.7 Statistical analysis

Analyses were conducted in SPSS ver. 22 (SPSS Inc., Chicago, IL). *P* < 0.05 was considered significant in all analyses.

#### 2.7.1 Effect of increased latrine coverage on transmission routes

We tested whether intervention villages compared to controls had less frequent detection of: (1) total and human fecal makers in any tested transmission route, and (2) target pathogens in community water sources. To test each pathway (i.e. sample type), except hands, we used Poisson regression (log-link) with generalized estimated equations (GEE), robust standard errors and exchangeable correlation structure to account for village-level clustering. Because fecal contamination on mothers' and children's hands was strongly correlated (Schriewer et al., 2015), mixed effects logistic regression with random intercepts for village and household (accounting for clustering at each level) was used to compare prevalence of hand contamination.

Concentration of total fecal markers was also compared between intervention and control villages. Non-detected samples were assigned half their SLOD value. Due to a skewed distribution with zero inflation, gene copy counts were collapsed into log_10_ categories (0–1 gc, 1–10 gc, 11–100 gc, etc. per mL or two hands) and analyzed using ordered logistic regression with robust standard errors (to account for village-level clustering). Finally, we examined village-level detection rates for fecal and pathogen contamination for differences attributable to increased sanitation coverage using negative binomial regression (details in Supplemental Material).

#### 2.7.2 Microbial contamination, exposure pathways and child diarrhea

We examined whether households with human and animal fecal contamination (indicated by MST markers) had increased risk of under-5 child diarrhea, based on the 7-day period prevalence of child diarrhea measured by the Sanitation Trial within 6 weeks of testing. Specifically, we tested whether increasing levels of household human or animal fecal marker contamination were associated with subsequent household child diarrhea in MST sampled households, using logistic regression with GEE, robust standard errors, and exchangeable correlation structure. Three levels of contamination were defined: (1) all tested pathways (i.e. SDW, mother's hands, child's hands) were negative for the target marker, (2) some (but not all) were positive, and (3) all were positive.

We also examined whether the extent or type of detected diarrhea pathogen contamination of a village's community drinking water or bathing sources was associated with subsequent village-level child diarrhea prevalence rates, based on the 7-day period prevalence for all under-5 children in the village measured by the Sanitation Trial within 6 weeks of testing. Specifically, we tested whether more frequent detection of pathogens in groundwater drinking and/or domestic pond sources was associated with increased child diarrhea prevalence in the village following detection, using negative binomial regression with a log link function and robust standard errors.

For these analyses, study villages with a Sanitation Trial diarrhea surveillance visit (Clasen et al., 2014) occurring within 6 weeks following the date of environmental sampling (n = 37 of 60 villages) were included on the basis that the incubation period for diarrheal pathogens in an exposed child is no longer than 6 weeks. Therefore, only one diarrhea surveillance observation per MST household in the household-level analysis, and per MST village in the village-level analysis, was used. See Supplemental Material for modeling details and descriptive statistics of dependent and independent variables.

## 3 Results

### 3.1 Human fecal contamination of community water sources, stored drinking water and hands

**Fig. 1:**
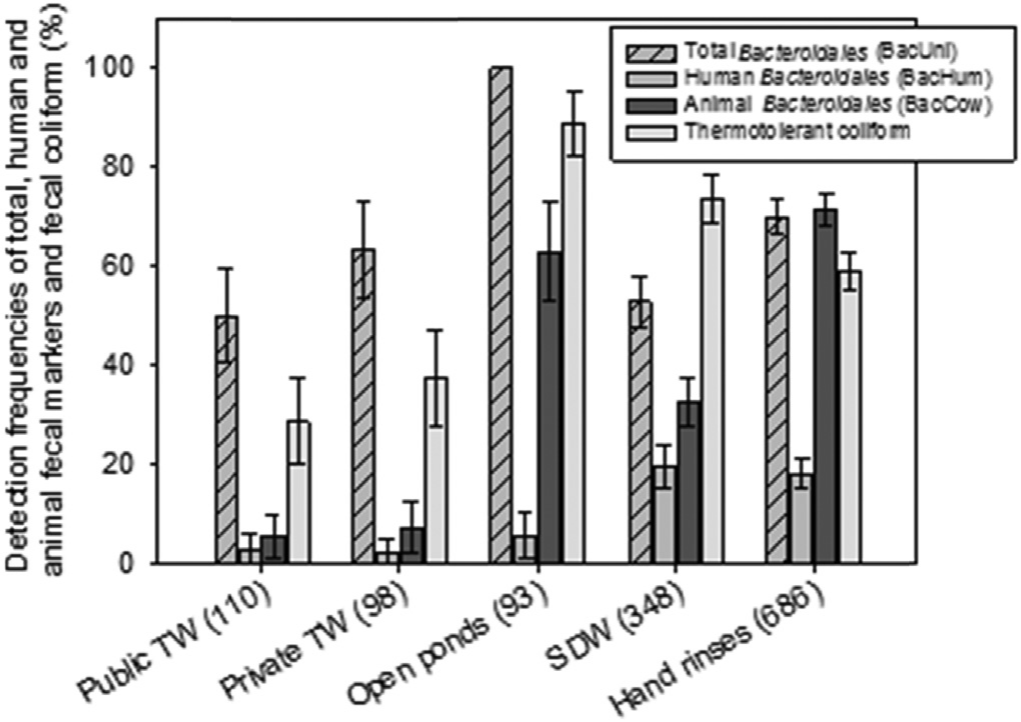
Detection frequencies of total, human, and domestic animal *Bacteroidales* and thermotolerant coliform (fecal coliform) in each exposure pathway (transmission route). Numbers in parenthesis refer to the number of water sources or household samples collected and tested. Error bars represents 95% confidence intervals.

Among community water sources, more than half of public (50.0%, 95%CI: 40.7–59.3%) and private tubewells (63.3%, 95%CI: 53.7–72.8%), and all ponds (100%) were positive for total fecal markers (BacUni) (Fig. 1). TTC were detected less often, in 28.6% (95%CI: 19.9–37.2%) and 37.2% (95%CI: 27.5–47.0%) of public and private tubewells, respectively, and 88.9% (95%CI: 82.4–95.4%) of ponds. Human fecal contamination (BacHum) of community water sources was rarely detected (2.8% [95%CI: 0.0–5.8%] of public and 2.1% [95%CI: 0.0–4.9%] of private tubewells, and 5.6% [95%CI: 0.8–10.3%] of ponds) (Fig. 1). Animal fecal markers (BacCow) were frequently detected in ponds (63.0%, 95%CI: 53.2–72.9%), but less frequently in tubewell drinking sources (6.3%, 95%CI: 3.0–9.7%).

In homes, total fecal contamination (BacUni) was detected in 52.9% (95%CI: 47.6–58.1%) of SDW and on 69.9% (95%CI: 66.5–73.4%) of hands; TTC was detected more frequently in SDW (73.6%, 95%CI: 68.9–78.4%) than on hands (58.9%, 95%CI: 55.1–62.7%). Detection of human fecal markers was similar in SDW (19.4%, 95%CI: 15.2–23.6%) and on hands (18.0%, 95%CI: 15.1–20.9%), while animal fecal markers were more frequently detected in both sample types (32.7% [95%CI: 27.7–37.6%] of SDW and 71.5% [95%CI: 68.1–74.9%] of hands) (Fig. 1). Considering SDW or hands detected with fecal markers as evidence of contamination in the home, more than 70% of households had detectable total and animal fecal contamination, whereas 35% had detected human fecal contamination. Fecal marker and TTC concentration results are reported in Supplemental Material (Tables S2 and S3).

### 3.2 Seasonal variability of fecal contamination

The prevalence of fecal contamination of community water sources did not differ between 2012 and 2013, except for TTC in public tubewells and animal fecal markers (BacCow) in ponds, for which detection rates were significantly lower in 2013 (Table S2, Supplemental Material). Unlike community water sources, household samples generally showed significantly less contamination in 2013 than in 2012 for each MST marker, except for human fecal markers in SDW (Table S3, Supplemental Material).

**Fig. 2:**
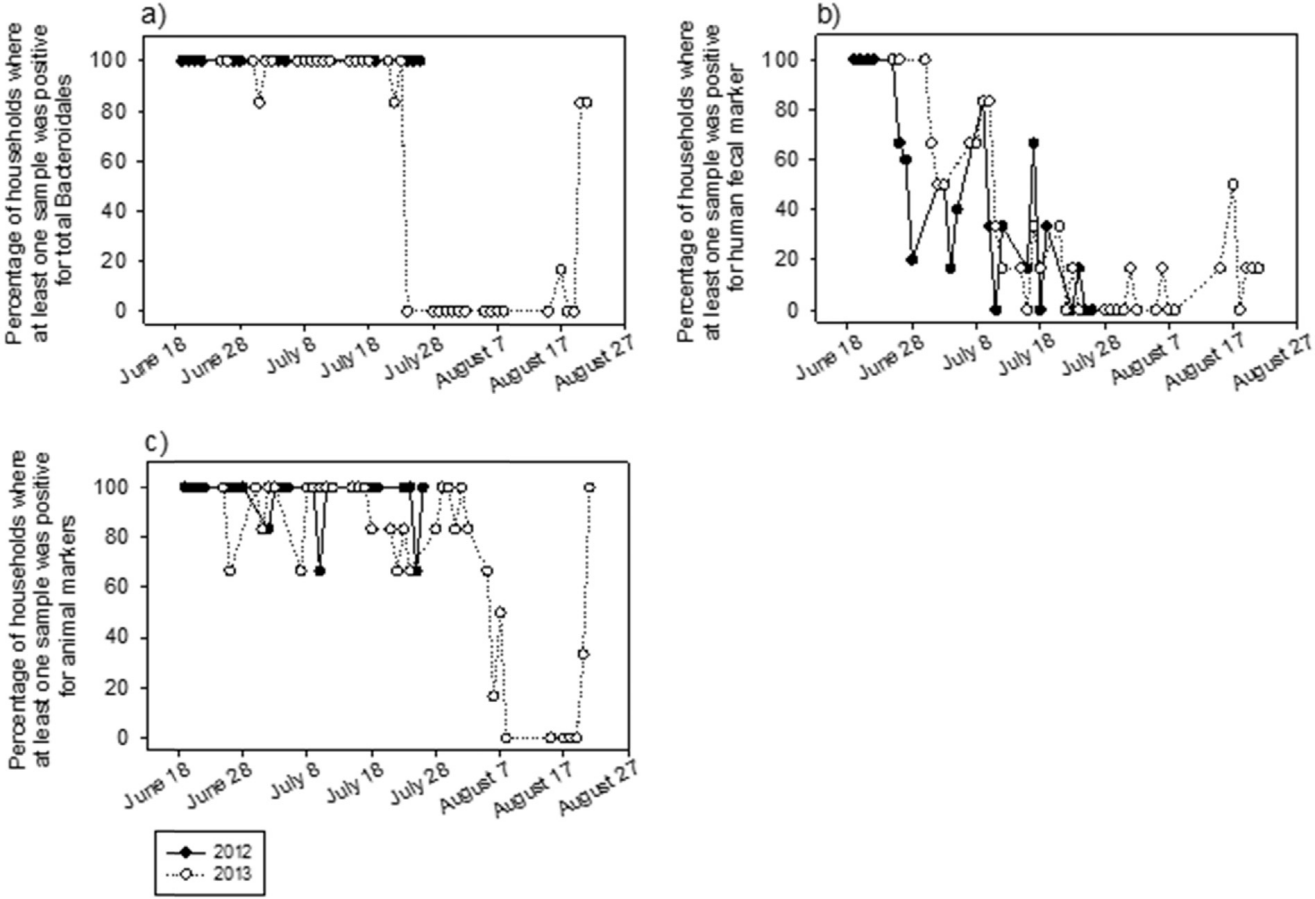
Temporal variability in the proportion of sampled households positive for (a) total, (b) human, and (c) domestic/livestock animal fecal markers in each village. Black dot and empty circle denote 2012 and 2013, respectively. Each circle is one village.

To further explore apparent seasonal differences in domestic domain fecal contamination, the temporal proportion of sampled households with detected contamination during the 2012 and 2013 monsoon sampling seasons was plotted for each MST marker (Fig. 2). Two interesting trends emerge. First, contamination rates in 2012 and 2013 were similar for all three fecal markers during the early part of the monsoon season (i.e. before July 28th), followed by a notable drop after July 28th, when sampling occurred in 2013 only. Second, rates of homes contaminated with total and animal fecal markers (Fig. 2a and c) were consistently at or near 100% both years during the early part of the monsoon season, whereas rates of homes contaminated with human fecal markers steadily declined from 100% to 0% as the season progressed in both years. Unlike the temporal patterns of fecal contamination in homes, rates of contamination of local groundwater sources showed no clear trend during the first part of the monsoon season in either year (Fig. S1, Supplemental Material). There is some evidence in the 2013 sampling season of reduced contamination after July 28th. Thus, much of the apparent difference in observed contamination between 2012 and 2013 seems to result from the longer 2013 sampling season which extended into and throughout the much wetter monsoon month of August.

### 3.3 Pathogen contamination of community water sources

**Fig. 3:**
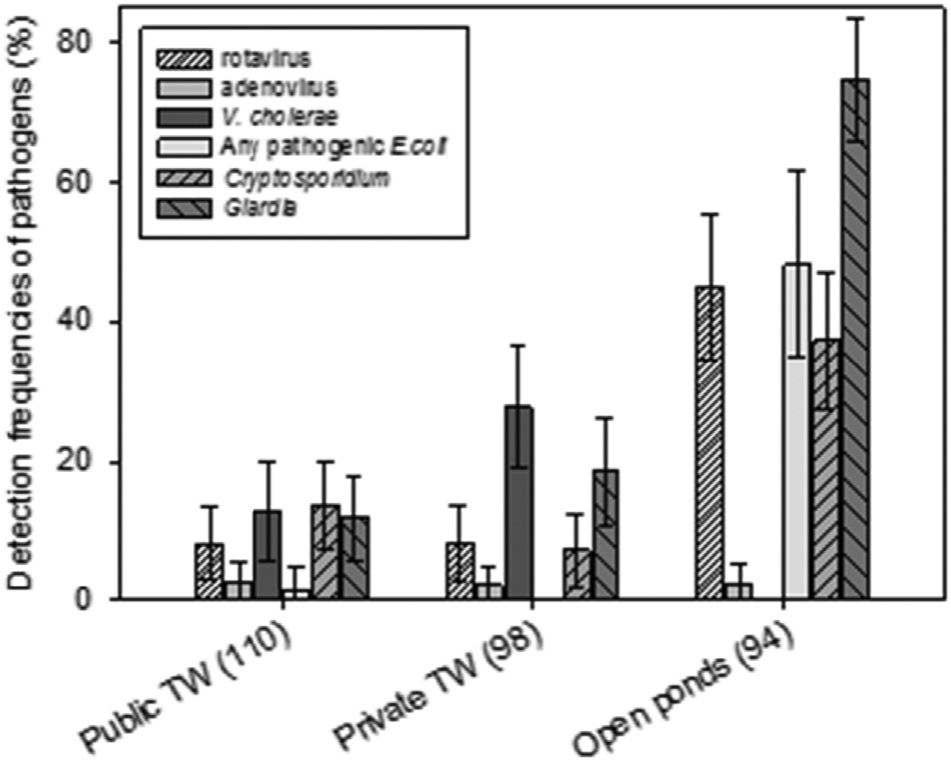
Detection frequencies of viral (rotavirus and adenovirus), bacterial (*Vibrio cholerae* and any of 7 tested pathogenic *Escherichia coli* virulence genes) and protozoan (*Cryptosporidium* and *Giardia*) pathogens in community water sources. Pathogenic *E. coli* was tested in the subset of 2013 sources only. Numbers in parenthesis refer to the number of sources sampled (once each) and tested. Error bars represent 95% confidence intervals.

A wide range of pathogens was detected in local groundwater sources used for drinking (Fig. 3). *Vibrio cholerae* (*ctxA* gene) was frequently detected in both public and private tubewells (12.8%, 95%CI: 6.6–19.1% and 27.6%, 95%CI: 18.7–36.4%, respectively). Protozoan pathogens were also detected with relatively high frequencies in these sources; 13.5% (95%CI: 7.2–19.9%) of public and 7.1% (95%CI: 2.0–12.2%) of private tubewells were positive for *Cryptosporidium*, while *Giardia* was detected in 11.7% (95%CI: 5.7–17.7%) of public and 18.4% (95%CI: 10.7–26.0%) of private tubewells. Among viral pathogens, rotavirus was most often detected (8.3% [95%CI: 3.1–13.4%] in public and 8.2% [95%CI: 2.7–13.6%] in private tubewells). Adenovirus 40/41 and seven pathogenic *E. coli* virulent genes were rarely detected in any tubewells (Fig. 3).

Compared to protected groundwater sources, as might be expected, pathogens were detected with greater frequency and/or at higher concentrations in community ponds, with one exception: *V. cholerae* was never detected (Fig. 3). Rotavirus, *Cryptosporidium*, *Giardia* and at least one of the seven pathogenic *E. coli* virulent genes were detected in 44.9% (95%CI: 34.6–55.3%), 37.2% (95%CI: 27.5–57.0%), 74.5% (65.7–83.3%) and 48.1% (34.8–61.7%) of ponds, respectively. Of the seven pathogenic *E. coli* virulent genes tested, *eaeA* (EHEC/EPEC) was most frequently detected, followed by *aggR* (EAEC) (detailed results in Table S6, Supplemental Material). Adenovirus 40/41 was rarely detected in ponds (Fig. 3), similar to groundwater sources, but at higher concentrations. Overall, 31.5% (95%CI: 22.9–40.2%) of public and 39.8% (95%CI: 30.1–49.5%) of private tubewells, and 89.4% (95%CI: 83.1–95.6%) of ponds were positive for at least one of five pathogens measured (excluding pathogenic *E. coli*). Pathogen concentrations are reported in Supplemental Material (Table S5).

### 3.4 Seasonal variability of pathogen contamination

Detection of each tested pathogen in public tubewells was significantly lower in 2013 than in 2012 (Table S5, Supplemental Material). Similarly, every pathogen was less frequently detected in private tubewells in 2013, but only differences for *V. cholerae* and *Giardia* were statistically significant (Table S5, Supplemental Material). Consistent evidence for reduced pathogen contamination rates in 2013 in surface ponds was not found. While detection of *Cryptosporidium* was significantly lower in 2013, for *Giardia*, it was also lower but the difference was not significant, whereas for rotavirus it was significantly higher and remained essentially unchanged for adenovirus and *V. cholerae*.

**Fig. 4:**
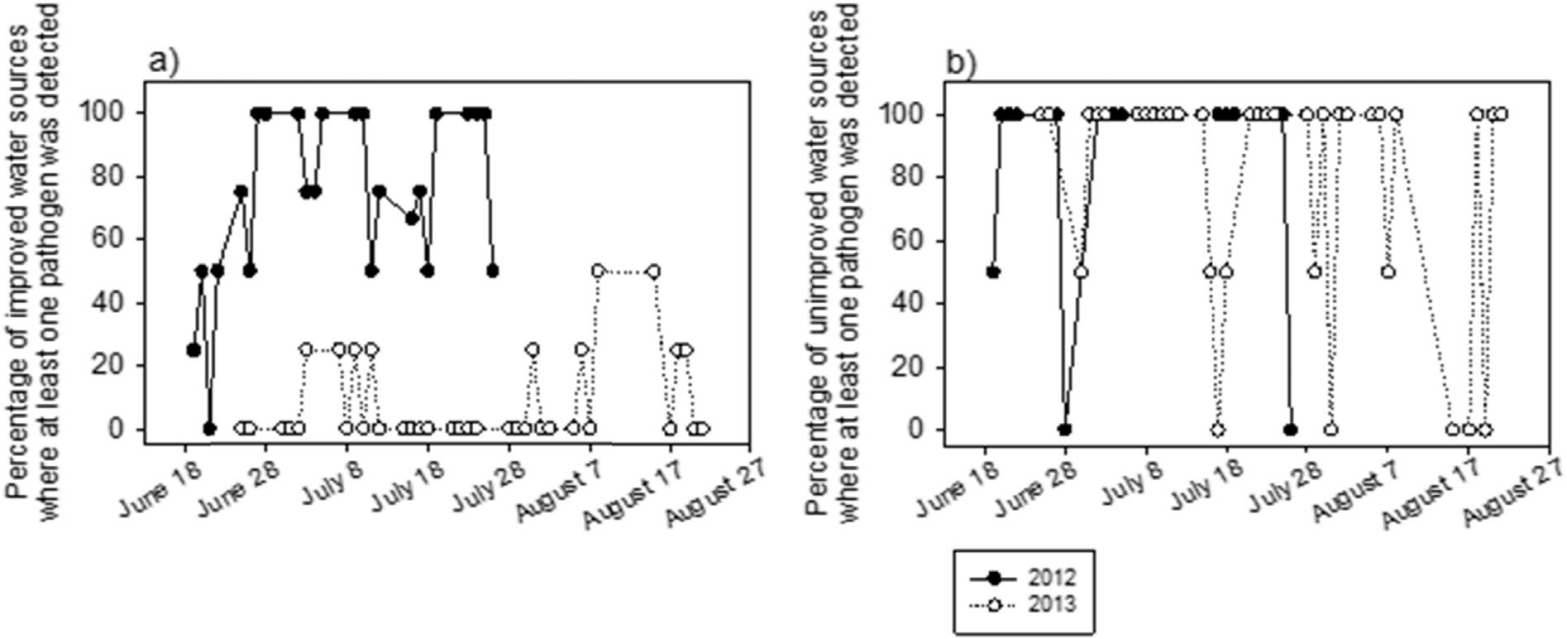
Temporal variability in the proportion of (a) improved (public and private tubewells) and (b) unimproved (pond) water sources positive for any of five pathogens (i.e. rotavirus, adenovirus, *V. cholerae*, *Cryptosporidium* or *Giardia*) in each village. Pathogenic *E. coli* was excluded because it was not measured in both years. Black circles and empty circles denote year of 2012 and 2013, respectively. Each circle is one village.

The proportion of a village's community water sources with at least one detectable pathogen, excluding pathogenic *E. coli*, was plotted to examine temporal patterns of exposure to pathogens via community water sources during the two monsoon seasons (Fig. 4; each day is one village). The percentage of a village's tested tubewells positive for any pathogen remained at or above 50% throughout the sampling period of 2012, except in two villages, whereas in 2013 it was consistently low, at or below 25%, except for some sporadic increases later in the season (Fig. 4a). In contrast, tested ponds showed no notable differences between years nor any temporal trend with respect to pathogen contamination (Fig. 4b). Regardless of time, most villages had ponds with at least one detectable pathogen measured, and only seven villages had ‘safe’ ponds (i.e. none of five pathogens, excluding pathogenic *E. coli*, detected).

### 3.5 Effect of increased latrine coverage on fecal contamination

**Fig. 5:**
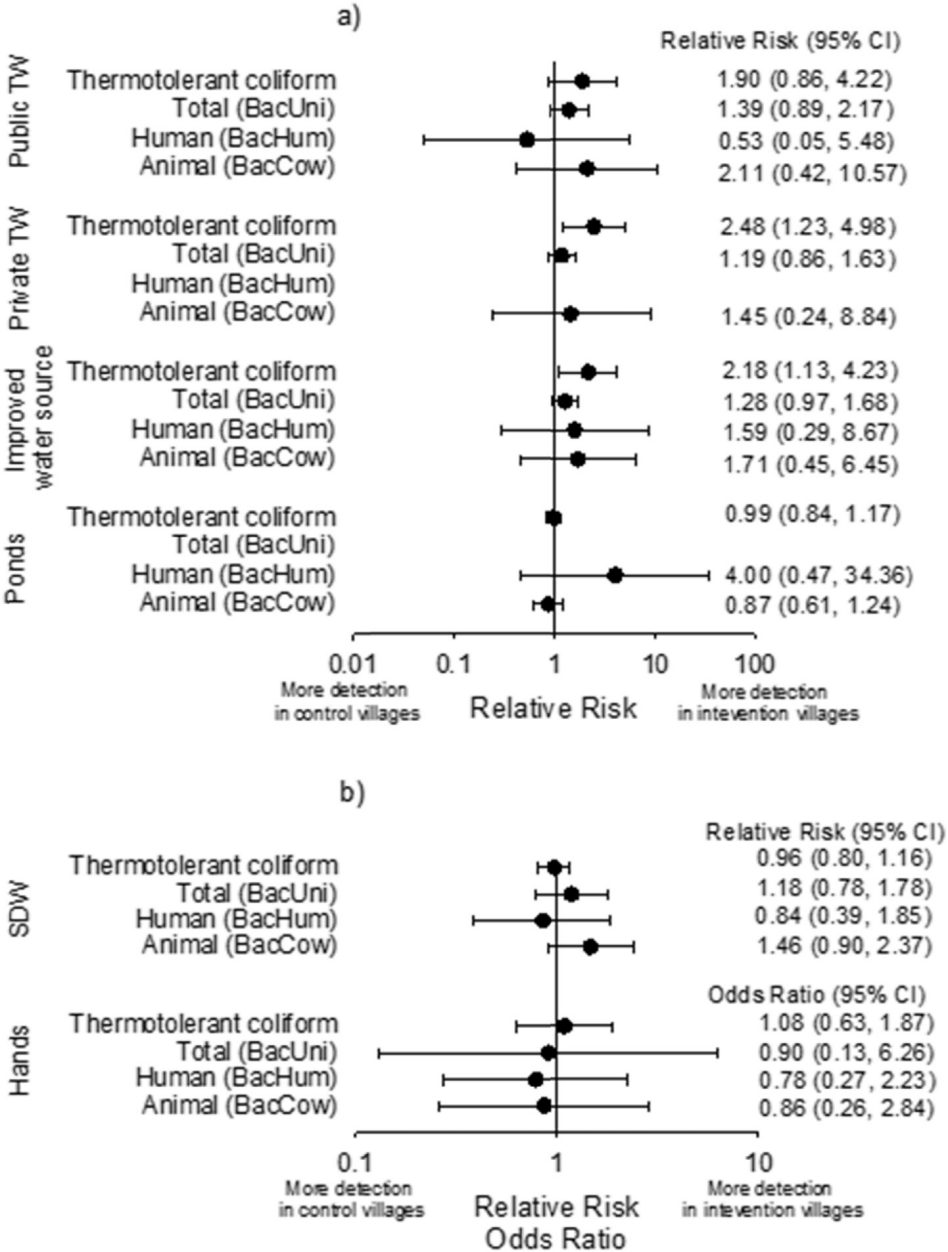
Forest plots of relative risks and odds ratios of detecting fecal markers in (a) community water sources and (b) homes in Sanitation Trial intervention over control villages. Relative risk for BacHum in private tubewells was not calculated due to the small number of detected tubewells (n = 2), while those for BacUni in open ponds could not be estimated because all sampled sources were positive. TW = tubewell, and SDW = stored drinking water.

There was no significant reduction between intervention and control villages in the prevalence of TTC, total, human or animal fecal contamination (Fig. 5), nor in log_10_ concentrations of total fecal markers (Table 1), for any public or domestic domain tested transmission pathway (i.e. any sample type). There was, however, suggestive evidence that intervention villages had more fecal contamination in their water sources. More frequent detection of fecal contamination as measured by both TTC and total *Bacteroidales* was found in drinking sources (i.e. public and private tubewells combined) in intervention compared to control villages (Relative Risk: 2.18, 95%CI: 1.13, 4.23, and 1.28, 95%CI: 0.97–1.68, respectively) (Fig. 5). Drinking sources in intervention villages also showed evidence of higher log_10_ levels of total *Bacteroidales* markers (ordered logistic regression Odds Ratio: 1.77, 95%CI: 0.97–1.68) (Table 1). A weak trend was also found of more human fecal contamination in ponds of intervention villages. However, the small number of sampled ponds with human contamination (n = 5) created a wide confidence interval (Relative Risk: 4.0, 95%CI: 0.47–34.5) (Fig. 5). There was also suggestive evidence that SDW in intervention villages was more likely to contain animal fecal markers (Relative Risk: 1.46, 95%CI: 0.90–2.37) (Fig. 5). No effect of increased latrine coverage was observed on the village-level proportion of tested households positive for total, human or animal fecal markers either (Relative Risk: 0.96, 95%CI: 0.72–1.29; 1.06, 95%CI: 0.65–1.73; and 1.07, 95%CI: 0.88–1.29, respectively).

### 3.6 Effect of increased latrine coverage on pathogen contamination

**Fig. 6:**
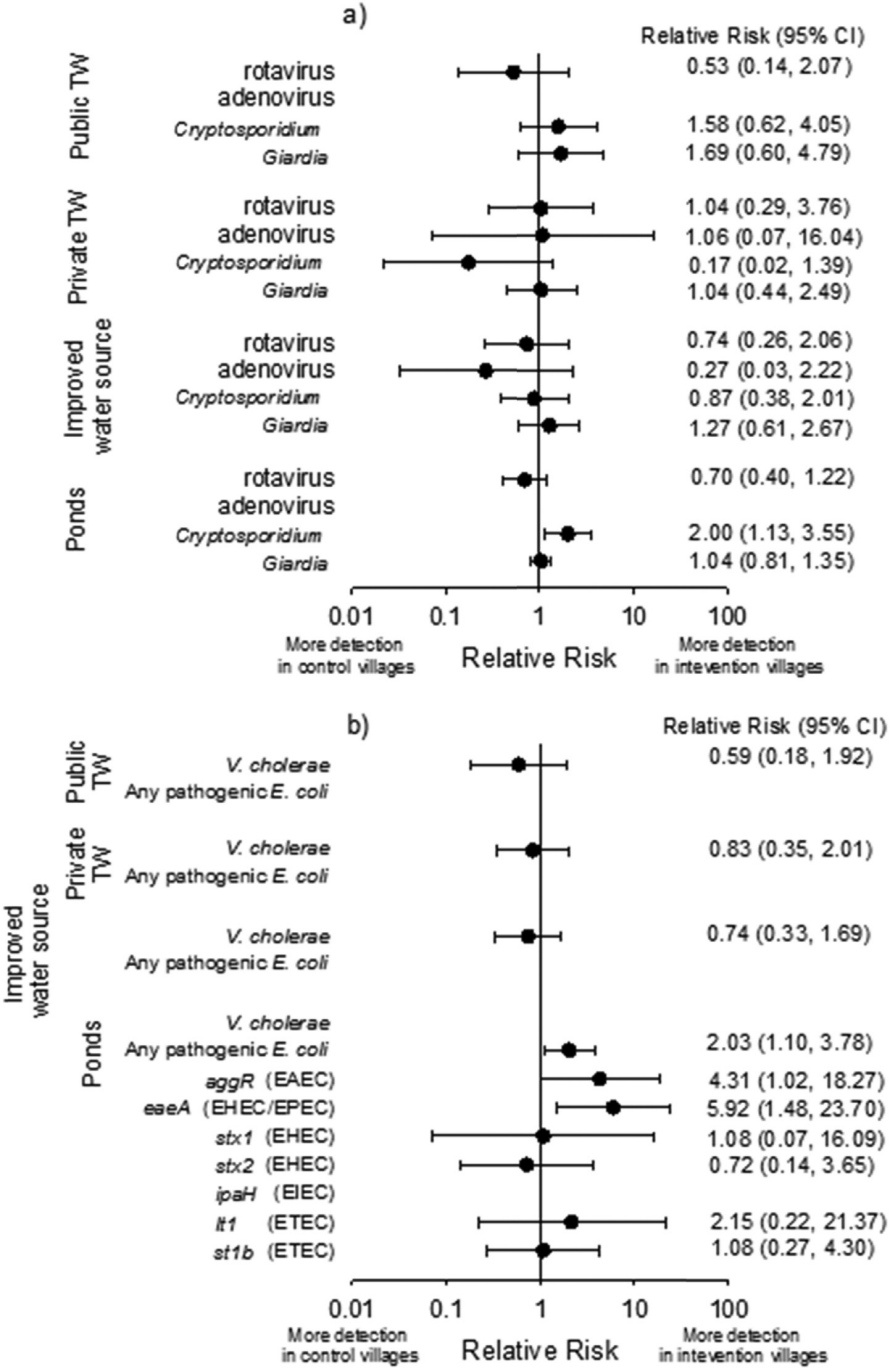
Forest plots of relative risks of (a) viral and protozoan and (b) bacterial pathogens in community water sources in Sanitation Trial intervention over control villages. Relative risks for adenovirus in public tubewells and ponds were not calculated due to the small number of detected sources (n = 2 and n = 3, respectively). Relative risks for pathogenic *E. coli* in public and private tubewells individually, and combined (i.e. improved water sources) were not calculated due to the small number of detected sources (n = 1). *V. cholera* was not detected in open ponds.

We found no evidence for reduced prevalence of enteric pathogens in protected groundwater drinking sources in intervention compared to control villages (Fig. 6). The proportion of tubewells contaminated with pathogens (excluding pathogenic *E. coli*) was similar (Relative risk: 0.84, 95%CI: 0.47–1.50). However, several pathogens were detected in surface water ponds at significantly higher rates in intervention than control villages. *Cryptosporidium* and pathogenic *E. coli*, specifically *aggR* and *eaeA* genes (commonly found in EAEC and EHEC/EPEC, respectively) were detected 2.00 (95%CI: 1.13–3.55), 4.31 (95%CI: 1.02–18.27) and 5.92 (95%CI: 1.48–23.70) times more often, respectively, in intervention than control village ponds.

### 3.7 Associations between fecal and pathogen contamination rates and child diarrhea

Levels of human and of animal contamination in the home were significantly associated with occurrence of reported child diarrhea within 6 weeks after household sampling (Table 2). Compared to households with no detected contamination of exposure pathways (all samples negative for the MST marker), households where all three pathways (samples) had detected human markers or detected animal markers showed 4.18 (95%CI: 1.30–13.46) and 4.54 (95%CI: 1.17–17.59) higher likelihood of having a child with reported diarrhea. We also found strong statistical evidence of a positive association between the proportion of a community's drinking water tubewells positive for any tested pathogen (excluding pathogenic *E. coli*) and child diarrhea prevalence in the community following exposure (Table 3). The proportion of ponds detected with any pathogen was not associated with an increased prevalence of child diarrhea (RR 0.96, 95%CI: 0.36–2.53). Further analysis of specific pathogens detected in tubewell drinking sources at village-level revealed that child diarrhea prevalence was 1.84 (95%CI: 1.19–2.85) times higher when *V. cholerae* was detected and 1.48 times higher (95%CI: 0.89–2.44) when *Giardia* was detected (Table 3). On the other hand, detection of rotavirus in ponds was associated with lower child diarrhea prevalence (RR 0.64, 95%CI: 0.41–1.02).

## 4 Discussion

### 4.1 Human fecal and pathogen contamination in study villages

#### 4.1.1 Improved groundwater drinking sources

More than 50% of tubewells had detectable fecal contamination measured by the total *Bacteroidales* fecal marker. We identified both humans (n = 4 TWs) and domestic animals (n = 13 TWs) as host sources of observed contamination, but were unable to identify the primary source of fecal contamination in many cases due to low levels of contamination. A recent systematic review documented widespread fecal contamination of drinking water including improved water sources in developing countries, especially in rural areas (Bain et al., 2014). Our results support these findings, and cast doubt on the assumption that improved water sources are microbiologically safe in estimating the 2010 global burden of disease (Lim et al., 2012).

Surprisingly, more than 30% of protected groundwater drinking sources had at least one detectable diarrheal pathogen among the five we tested consistently. *Vibrio cholerae* was detected most frequently (19.8%) at very low concentrations (geometric mean of positives 3 gc/mL), although no cholera outbreaks were reported in the study communities during sampling in 2012 and 2013. Flood-associated cholera outbreaks have been reported in some parts of Odisha (Chhotray et al., 2002, Kumar et al., 2009, Pal et al., 2010)*. V. cholerae* is known to be present naturally in aquatic environments associated with copepod species of zooplankton (Colwell, 1996). As we never detected *V. cholerae* in freshwater ponds, and detection rates were greater in shallow private tubewells than in deep public tubewells, one plausible mechanism for *V. cholerae* contamination of subsurface groundwater in the study area is the aquifer intrusion of estuary or coastal water carrying plankton (Constantin de Magny et al., 2008). Other pathogens, including rotavirus, *Cryptosporidium* and *Giardia*, were detected in 8.2–14.8% of protected groundwater sources, presenting significant drinking water microbial risks in these communities. Indeed, we found a significantly increased village prevalence of child diarrhea in the 6 weeks following sampling for each additional tubewell detected with a tested diarrheal pathogen. Pathogens in protected groundwater sources, commonly used for drinking without disinfection in developing countries, have been reported elsewhere. In rural Bangladesh, shallow tubewells were found to be contaminated with multiple pathogens including pathogenic *E. coli*, *Shigella* spp., *Vibrio* spp. and rotavirus (Ferguson et al., 2012). Viral contamination with rotavirus and adenovirus was detected in 14% and 2% of wells, respectively, in rural Benin, West Africa, where the presence of latrines within a 50 m radius appeared to be a risk factor (Verheyen et al., 2009).

We did not observe any notable temporal pattern in the proportion of tubewells positive for total fecal markers, or any pathogens, during the monsoon season. In contrast, the annual difference in pathogen detection rates was significant, with much less detection in 2013. Across Sanitation Trial villages, child diarrhea prevalence varied over time, and lower child diarrhea prevalence was observed during 2013 than during 2012 (Clasen et al., 2014), consistent with the between-years difference in pathogen contamination observed in improved groundwater sources. A review of long-term child diarrhea prevalence data in Pakistan also found large variability in prevalence rates from year to year, indicating highly variable exposure to gastrointestinal pathogens between years (Luby et al., 2011). Dilution effects due to heavier rainfall in 2013 might partially explain less frequent detection of pathogens in 2013. Examining local station daily rainfall data in June and July of 2012 and 2013 suggests that rainfall in 2013 was marginally significantly greater in one study block (Puri) (7.9 mm/day in 2012 *vs.* 13.3 mm/day in 2013, Mann-Whitney *U* test, p = 0.07), but not in three other study blocks (Gop, Pipili and Nimapara).

#### 4.1.2 Domestic surface water sources

Despite continued open defecation across study areas, human fecal markers were rarely detected in community ponds, likely due to a relatively high concentration of human feces required for marker detection (Odagiri et al., 2015, Schriewer et al., 2015). More than half of ponds had detectable domestic/livestock animal fecal contamination with patterns persisting during the initial monsoon season in both years, indicating extensive animal fecal loading in study communities. Despite infrequent detection of human fecal markers, we frequently detected multiple diarrheal pathogens in ponds, including *Giardia*, pathogenic *E. coli* virulent genes (especially, *eaeA* (EHEC/EPEC), and *aggR* (EAEC) genes), rotavirus, *Cryptosporidium* and adenovirus 40/41 (in order of frequency), throughout our monsoon sampling period in both 2012 and 2013. We did not find any significant correlation between detection of human-associated fecal markers and detection of pathogens in either improved groundwater drinking sources or domestic surface water sources (data not shown) due to very few samples positive for human fecal markers compared to the numbers of samples positive for pathogens in this study. Poor correlation between detection of human-associated *Bacteroidales* genetic markers and pathogens can arise due to differences in environmental persistence and survival, differences in assay performance, and presence of pathogen strains or genotypes of non-human origin (i.e. zoonotic pathogens) (Fremaux et al., 2009, Schriewer et al., 2010).

Recent research in nearby Bangladesh identified EAEC, EPEC and rotavirus as among the most frequent probable contributors to early childhood diarrhea and found that infants were exposed to multiple pathogens as early as the first month of life (Taniuchi et al., 2013). The GEMS research on causes of child diarrhea identified rotavirus and *Cryptosporidium* as the two most important etiological agents in India (Kotloff et al., 2013). Tested ponds were public and used for multiple purposes including daily bathing, swimming, brushing teeth, washing utensils, laundry and anal cleansing after defecation by a majority of the local population in our rural study communities. Their use presents an under-studied and potentially under-recognized microbial health risk through direct or indirect ingestion of pathogenically contaminated pond water.

Of pathogens detected in open ponds, shiga-toxin producing EHEC, *Cryptosporidium* and *Giardia* have been reported to include zoonotic species (Baldursson and Karanis, 2011, Synge, 2000). Study area livestock animals appear to have a high prevalence of *Cryptosporidium* and *Giardia* (Daniels et al., 2015). Moreover, according to genotyping results of some of our water samples, a zoonotic type of *Giardia* was identified (Ibid). It is also important to note that we identified both human and animal rotavirus in open ponds based on sequencing results from qPCR amplicons of our rotavirus positive pond samples (data not shown). Despite known host-specific infection of rotavirus, re-assortment of animal rotavirus with human strains could produce a novel strain posing human health risks (Afrad et al., 2013, Martella et al., 2010). Together with high prevalence of domestic animal fecal contamination and detection of possible zoonotic pathogens in open ponds, diarrhea risks associated with daily domestic activities in ponds need further investigation as improved household sanitation is unlikely to alter exposures to zoonotic pathogens.

#### 4.1.3 Home environment (stored drinking water and hands)

Widespread fecal contamination in households was observed. Total, human and animal fecal markers were detected in 74.2%, 36.2% and 82.2% of households, indicating both human and animal fecal contamination and exposure are occurring in households. Human contamination detected on mothers' and children's hands was highly correlated and each was significantly correlated with human contamination detected in SDW; similar correlations were found between mothers' hands, children's hands, and SDW for detected animal fecal contamination in this setting (Schriewer et al., 2015).

The temporal proportion of households positive for each fecal marker revealed that villages with ‘clean’ households (i.e. no detection of any fecal markers in any household tested) were clustered in time during the later rainy season in 2013 (after July 28th). Research in rural Ecuador found a negative association between rainfall and microbial contamination of household stored water during the rainy season, measured by *E. coli*, possibly due to reduced water source contamination, increased contaminant dilution, and/or higher turnover rate of household stored water (Levy et al., 2009). If mothers' and children's contaminated hands play a role in the deterioration of the microbiological quality of SDW (Schriewer et al., 2015), fecal contamination of the home environment may have declined during the later rainy season in our study setting through dilution, and/or through weather-induced changes in mothers' daily routines and behaviors (e.g. switching from open defecation to latrine use, due to inundation of open land and heavy rainfall [P. Routray personal communication]) such that mother's hand contamination was reduced. Research in Tanzania found that touching environmental surfaces can quickly result in mothers' hand fecal contamination (Pickering et al., 2011). Another Tanzania study demonstrated that soil collected from various locations within a household including food preparation areas was contaminated with total and human *Bacteroidales* as well as pathogens (Pickering et al., 2012). Hence, one possible cause of fecal contamination on mothers' hands could be environmental surfaces in the home including the soil, for which sufficient continuous rainfall may be needed before accumulated fecal contamination from the preceding dry season months (January to June) is diluted or washed out. Further investigation is needed to understand the causes and dynamics of household fecal contamination over time, including prior to and within the rainy season.

### 4.2 Effect of increased latrine coverage on exposure pathways

We found no evidence that the increase in functional latrine coverage achieved by the intervention (+27 points) reduced human fecal contamination of community surface or groundwater water sources, or of SDW or hands in the home. Consistent with finding no reduction in human fecal contamination, there was also no positive impact on prevalence of enteric pathogens in improved drinking water sources. These results help explain the Sanitation Trial finding that the intervention had no effect on diarrheal diseases among children under five or among all ages (Clasen et al., 2014). There are several possible explanations for the lack of impact on human fecal contamination. First, the coverage difference between intervention and control groups was relatively small (10% *vs*. 38%). While 94% of households with a functional latrine were using it, usage may have been inconsistent and infant and young child feces were likely to be left in the open (Clasen et al., 2014, Jenkins et al., 2014). Accumulated environmental contamination from years of open defecation may take more time to disappear and our sampling occurred between 6 and 20 months after the intervention ended. However, a stratified analysis by year to explore whether lower groundwater pathogen contamination rates observed in 2013 compared to 2012 could be attributed to the intervention failed to find supportive evidence (See Fig. S2, Supplemental Material). Alternatively, if fecal loading from sources spread across a much larger spatial scale is mainly responsible for the observed fecal and pathogen contamination of exposure pathways in study villages, then within-village sanitation improvements alone would have little impact.

Contrary to expected positive impacts of increased sanitation access, we found suggestive evidence of an increase in both the detection and concentration of total fecal markers and in the detection of TTC in groundwater in intervention villages (Table 1, Fig. 5a). When stratified by year, a nearly significant increase in detection of total fecal makers in groundwater drinking sources due to the intervention was detected in 2013 but not in 2012 (see Fig. S3, Supplemental Material), a pattern that is consistent with a rise in sub-surface contamination with human feces from pour flush soak pits of newly installed latrines in intervention villages for which human fecal loading would have first started to accumulate in 2012. Detailed spatial analyses of latrine pit locations, ages, and usage relative to individual tubewells would be required to confirm potential negative impacts of leaching from household latrines on fecal and pathogen contamination of groundwater in this setting.

Interestingly, we found significantly greater prevalence of *Cryptosporidium* and two pathogenic *E. coli* virulent genes, *aggR* (EAEC) and *eaeA* (EHEC/EPEC), in ponds of intervention villages. Univariate analyses indicate that the occurrence of an extreme daily rainfall event (defined as rainfall exceeding the 90th percentile of total daily rainfall) within two days of sampling was significantly associated with increased odds of a pond being positive for *Cryptosporidium* and *aggR* gene (data not shown). Despite our sampling strategy to minimize the influence of spatial and temporal confounding by paring each intervention village with a unique nearest control village, the proportion of sampled ponds located in a study block experiencing at least one extreme rainfall event in the two days prior to sampling was significantly higher in intervention than control villages (χ^2^ p < 0.05). This partially explains the greater prevalence of these pathogens in intervention villages (Young et al., 2015). Additionally, heterogeneity of pond sampling site factors such as presence of animals at the time of sampling could be another contributing reason. Further detailed investigations with longitudinal monitoring would be necessary to better track temporal changes in the causes and sources of pathogen contamination in ponds.

### 4.3 Associations between child diarrhea and human and animal fecal contamination and pathogen detection

Our findings that the level of human and of animal fecal contamination of exposure pathways in households showed significant associations with subsequent child diarrhea suggests that exposure to not only human fecal but also animal fecal contamination may increase the risk of child diarrhea in this setting. This is consistent with our detecting potential zoonotic diarrhea pathogens widely in the study communities and high rates of local animal pathogen shedding in the study area from known zoonotic hosts (Daniels et al., 2015). We found that when more of a village's tubewell drinking sources were detected with a diarrheal pathogen, significantly higher child diarrhea prevalence was observed within 6 weeks after testing. Specifically, detection of *V. cholerae* in drinking sources was associated with significantly elevated prevalence of child diarrhea. However, the ingested *V. cholerae* in contaminated drinking water may itself not have caused the elevated diarrheal risks as it generally requires a large number of cells (10^6^ to 10^11^ cells) to infect a host successfully (Schmid-Hempel and Frank, 2007), and the concentrations we found were considerably lower (arithmetic mean concentration of positives: 10^4^ gc per L). Additionally, no cholera outbreak was reported during our sampling periods. Instead, other unmeasured risks for child diarrhea may occur when *V. cholerae* is present in local groundwater in this setting which due to our cross-sectional design we could not identify. Interestingly, detection of rotavirus in ponds was nearly significantly associated with a reduced child diarrhea prevalence rate in the village. A cohort study in India has shown that severity of diarrhea was significantly reduced between second and third infections of rotavirus (Gladstone et al., 2011), supporting the hypothesis that frequent exposures to natural rotavirus in ponds may help protect against human rotavirus infection among children under five.

### 4.4 Limitations

Every effort was made to include the best available assays for microbial source tracking to minimize the detection of non-intestinals, that is, environmental strains of *Bacteroidales* that do not have a recent fecal origin. This was achieved by first validating assays for the location and habitat studied in our study (Odagiri et al., 2015). Worldwide, there are only two reports of detection of non-intestinals using the general BacUni assay. A study on alpine soils in Austria found false positives for BacUni at concentrations close to the detection limit and several orders of magnitude lower than concentrations in feces (Vierheilig et al., 2012). Several general fecal *Bacteroidales* markers, including BacUni, were tested in Dutch groundwater and tap water (van der Wielen and Medema, 2010). The authors reported that 9 out of 11 samples were positive for BacUni but no data were presented, and hence it is impossible to gauge whether concentrations were close to the detection limit. No human- or ruminant-associated fecal MST assays for *Bacteroidales* were positive in that study. We also searched available databases for positive detection of non-fecal *Bacteroidales* sequences using BacUni primers and probe. To this end plasmid sequences containing the target regions plus intermittent sequences in the 16S rRNA gene were aligned against the Nucleotide Collection (nt/nr) database using the BLAST homepage (http://blast.ncbi.nlm.nih.gov). Of the 100 top hits, 95 were for human feces, one was in an anaerobic reactor treating wastewater, one was from a cow rectum swab, one was from pig feces, one from the rhizosphere of soil, and one from an air sample. Hence the assay is highly specific for fecal material with only two environmental samples, one from the rhizosphere and one from air, giving false positive results. The 100 sequences in the database had been submitted from the following countries: China, France, India, Japan, and USA. Overall we conclude that the BacUni assay is an excellent tool for the detection of fecally derived *Bacteroidales* in India and other regions in the world that have been studied by independent research groups.

The prevalence of human fecal contamination in household samples is likely underestimated because of the lower BacHum sensitivity on individual human fecal samples and amount of target markers per unit mass of fecal material, compared to that for the domestic/livestock animal fecal assay, BacCow (Odagiri et al., 2015). According to Odagiri et al., human-associated BacHum fecal markers were detected in 40% of individual human fecal samples, but in 100% of sewage samples in India. Importantly, the mean concentration (per wet gram) of BacHum markers in pure fresh human fecal samples was two orders of magnitude lower than that of BacCow markers in pure animal fecal samples (Odagiri et al., 2015), indicating that significantly more human feces than domestic animal feces are necessary in a sample to detect human fecal contamination in the environment in this setting. Different detection limits for each sample type also reduce ability to directly compare detection rates across pathways, requiring careful consideration of detection lower limits and detected concentrations, due to different sample collection volumes, sample processing, and sample matrix effects.

It should be noted that pathogen qPCR and IMS-DFA results do not yield any information on infectivity or viability of cells. Pathogenic potential of bacteria was measured using qPCR and PCR. The assays which detected pathogenicity genes were validated prior to use against fresh diarrheal stool samples and positive isolates obtained from clinically positive diarrhea patients in Odisha, India (see Supplemental Material for details). Cultivation-based methods were not employed because field conditions and local laboratory facilities for enumeration were not available. Hence confounding may have affected some of the analyses presented here. For example, association between child diarrhea and the preceding level of human and animal fecal exposure in the home or pathogen exposure in drinking water may have been influenced by these villages having higher general exposure to feces and pathogens for socio-economic reasons which we were unable to test. However, biological plausibility lends support to the microbial exposure-to-disease associations we found and are consistent with lack of any health impact in the Sanitation Trail (Clasen et al., 2014). We did not have sufficient data to explore this more fully. In addition, other microbial indicators could have been used that may have led to stronger associations with illness. For example, additional benefits may have been gained from measuring *Clostridium perfringens* spores which may better describe recent human fecal contamination in subtropical regions (Fujioka and Shizumura, 1985). Future work should involve quantitative microbial risk assessment to confirm the validity of the secondary associations we found between village-level drinking water pathogen contamination and child diarrhea prevalence.

## 5 Conclusions

• Extensive human and animal fecal contamination observed in homes, which was associated with subsequent child diarrhea, points to the need for interventions beyond improving household sanitation, to reduce domestic domain microbial contamination and exposures.
• Observed widespread animal contamination in both the public and domestic domains, together with detection of zoonotic types of pathogens, point to a need for better animal excreta management to reduce potential health risks in rural communities.
• Pathogen detection in protected groundwater sources poses a significant waterborne microbial health risk for rural tubewell users in the region, while continued use for personal and domestic hygiene of community ponds highly contaminated with multiple pathogens presents an under-appreciated and unstudied health risk.
• A 27 percentage point increase in improved sanitation access at village level did not reduce detectable human fecal and pathogen contamination in this setting. Rather the possibility of increased microbial contamination of local groundwater drinking sources through sub-surface leaching from newly installed pour-flush latrine pits cannot be ignored and requires further detailed investigations.
• The proportion of village groundwater drinking sources positive for one or more of 5 diarrheal pathogens was associated with elevated child diarrhea prevalence observed within 6 weeks after detection. Specifically, detection of *V. cholerae* was significantly associated with an increased relative risk of child diarrhea in the ensuing 6 weeks.
• Rotavirus prevalence in community ponds showed potential protection against child diarrhea possibly though natural rotavirus exposures on children.

## Figures and Tables

**Table 1:** Effect of intervention on log_10_ levels of total *Bacteroidales* markers in community water sources, stored drinking water (SDW) and on hands.

Sample type	Median^a^ Log 10 (gc/mL or gc/two hands)		Adjusted^b^ OR^c^	95%CI	*p*-value
Intervention	Control
Public TW	1.12	0.50	2.05	0.88–4.78	0.10
Private TW	1.21	1.08	1.52	0.7–3.29	0.29
Improved drinking water sources (private and public TW)	1.13	0.80	1.77	0.97–3.24	0.07
Open ponds	4.10	3.95	1.23	0.55–2.77	0.62
SDW	1.01	−0.12	1.36	0.61–3.03	0.46
Hands	4.91	4.87	0.91	0.43–1.92	0.81

a A half sample detection limit (SLOD) was assigned when the sample was below SLOD.

b Adjusted for village-level clustering using Generalized Estimated Equation (GEE) with robust estimate errors.

c Odds ratio of being in a higher log level of total *Bacteroidales* calculated by ordered logistic regression.

**Table 2:** Association between levels of human and of animal fecal contamination of exposure pathways^a^ detected in the home and household under-5 child diarrhea prevalence^b^ within 6 weeks after sampling.

Tested variables	Number of households	Odds ratio	95% CI		*p*-value
*Level of human fecal contamination in a household:*					
All pathways (sample types)^c^ negative for human fecal marker	110	Ref.			0.044
Some pathways positive for human fecal marker	53	1.52	0.52	4.43	0.44
All pathways were positive for human fecal marker	11	4.18	1.3	13.46	0.02
*Level of animal fecal contamination in a household:*					
All pathways (sample types)^c^ negative for animal fecal marker	28	Ref.			0.013
Some pathways positive for animal fecal marker	92	1.58	0.42	5.97	0.50
All pathways positive for animal fecal marker	54	4.54	1.17	17.59	0.03

a Three levels of contamination were defined: (1) all 3 pathways (i.e. sample types: SDW, mother's HR, child HR) were negative, (2) some but not all were positive, or (3) all were positive. Human and animal fecal contamination was measured by the BacHum and BacCow assays, respectively.

b Household 7-day recall period prevalence for under-5 child diarrhea observed in the Sanitation Trial (Clasen et al., 2014) measured between 1 and 6 weeks after domestic domain environmental sampling for MST markers in the household.

c Stored drinking water (SDW), mother's hands (mother's HR), and children's hands (child HR).

**Table 3:** Association between village-level (1) proportion of tested improved community groundwater drinking water sources (tubewells), (2) proportion of tested domestic surface water sources (public ponds) positive for any tested diarrheal pathogen (rotavirus, adenovirus, *V. cholerae*, *Cryptosporidium* or *Giardia*), and (3) detection of specific diarrheal pathogen in improved drinking water tubewells and in community ponds, with observed village-wide under-5 child diarrhea prevalence^a^ within 6 weeks after sampling.

Tested variables	Number of villages	Relative risk	95% CI		*p*-value
Village-level proportion of tested improved drinking water sources (i.e. tubewells) positive for any of five tested pathogens	37	2.13	1.25	3.63	<0.01
Village-level proportion of tested surface water sources (i.e. pubic ponds) positive for any of five tested pathogens	36	0.96	0.36	2.53	0.93
*Pathogen detected in any tested tubewell water source in the village:*					
Rotavirus (Y/N)	37	0.92	0.53	1.62	0.78
Adenovirus (Y/N)	37	1.28	0.68	2.43	0.45
*V. cholerae* (Y/N)	37	1.84	1.19	2.85	<0.01
*Cryptosporidium* (Y/N)	37	1.18	0.72	1.92	0.52
*Giardia* (Y/N)	37	1.48	0.89	2.44	0.13
Any pathogenic *E. coli* (Y/N)	22	–b			
*Pathogen detected in any tested pond water source in the village:*					
Rotavirus (Y/N)	36	0.64	0.41	1.02	0.06
Adenovirus (Y/N)	36	–b			
*V. cholera* (Y/N)	36	–c			
*Cryptosporidium* (Y/N)	36	1.09	0.67	1.80	0.73
*Giardia* (Y/N)	36	1.22	0.60	2.48	0.59
Any pathogenic *E. coli* (Y/N)	22	1.11	0.58	2.12	0.75

a 7-day recall period prevalence among all under-5 children in the village as measured between 1 and 6 weeks after sampling by the Sanitation Trial (Clasen et al., 2014). Only 37 of 60 study villages had a diarrhea surveillance observation visit between 1 and 6 weeks of sampling. Of these 1 did not have any public ponds, and only 22 were sampled in 2013 when pathogenic *E. coli* was measured.

b Only one sample was positive.

c All samples were negative.

## References

[bib1] Afrad M.H., Matthijnssens J., Moni S., Kabir F., Ashrafi A., Rahman M.Z., Faruque A.S., Azim T., Rahman M. (2013). Genetic characterization of a rare bovine-like human VP4 mono-reassortant G6P[8] rotavirus strain detected from an infant in Bangladesh. Infect. Genet. Evol..

[bib2] Bain R., Cronk R., Hossain R., Bonjour S., Onda K., Wright J., Yang H., Slaymaker T., Hunter P., Pruss-Ustun A., Bartram J. (2014). Global assessment of exposure to faecal contamination through drinking water based on a systematic review. Trop. Med. Int. Health.

[bib3] Baldursson S., Karanis P. (2011). Waterborne transmission of protozoan parasites: review of worldwide outbreaks – an update 2004-2010. Water Res..

[bib4] Bambic D., MacBride G., Miller W., Stott R., Wuertz S. (2011). Quantification of Pathogens and Sources of Microbial Indicators for QMRA in Recreational Waters.

[bib5] Blackstone G.M., Nordstrom J.L., Bowen M.D., Meyer R.F., Imbro P., DePaola A. (2007). Use of a real time PCR assay for detection of the ctxA gene of *Vibrio cholerae* in an environmental survey of Mobile Bay. J. Microbiol. Methods.

[bib6] Boisson S., Sosai P., Ray S., Routray P., Trondel B., Schmidt W.P., Bhanja B., Clasen T. (2014). Promoting latrine construction and use in rural villages practicing open defecation: process evaluation in connection with a randomised controlled trial in Orissa, India. BMC Res. Notes.

[bib7] Chhotray G.P., Pal B.B., Khuntia H.K., Chowdhury N.R., Chakraborty S., Yamasaki S., Ramamurthy T., Takeda Y., Bhattacharya S.K., Nair G.B. (2002). Incidence and molecular analysis of *Vibrio cholerae* associated with cholera outbreak subsequent to the super cyclone in Orissa, India. Epidemiol. Infect..

[bib8] Clasen T., Boisson S., Routray P., Cumming O., Jenkins M., Ensink J.H., Bell M., Freeman M.C., Peppin S., Schmidt W.P. (2012). The effect of improved rural sanitation on diarrhoea and helminth infection: design of a cluster-randomized trial in Orissa, India. Emerg. Themes Epidemiol..

[bib9] Clasen T., Boisson S., Routray P., Torondel B., Bell M. (2014). Effectiveness of a rural sanitation programme on diarrhoea, soil-transmitted helminth infection, and child malnutrition in Odisha, India: a cluster-randomised trial. Lancet Glob. Health.

[bib10] Colwell R.R. (1996). Global climate and infectious disease: the cholera paradigm. Science.

[bib11] Constantin de Magny G., Murtugudde R., Sapiano M.R., Nizam A., Brown C.W. (2008). Environmental signatures associated with cholera epidemics. Proc. Natl. Acad. Sci. U. S. A..

[bib12] Daniels M., Smith W.A., Shrivastava A., Sahu P., Odagiri M., Misra P.R., Panigrahi P., Suar M., Clasen T., Jenkins M.W. (2015). Cryptosporidium and Giardia in humans, domestic animals, and village water sources in coastal Odisha, India. Am. J. Trop. Med. Hyg..

[bib13] Devamani C., Norman G., Schmidt W.P. (2014). A simple microbiological tool to evaluate the effect of environmental health interventions on hand contamination. Int. J. Environ. Res. Public Health.

[bib14] Eaton D.A., Clesceri S.L., Rice W.E., Greenberg E.A., Franson H.A.M. (2012). Standard Methods for the Examination of Water and Wastewater.

[bib15] Ferguson A.S., Layton A.C., Mailloux B.J., Culligan P.J., Williams D.E., Smartt A.E. (2012). Comparison of fecal indicators with pathogenic bacteria and rotavirus in groundwater. Sci. Total Environ..

[bib16] Fujioka R.S., Shizumura L.K. (1985). Clostridium perfringens, a reliable indicator of stream water quality. J. Water Pollut. Control Fed..

[bib17] Fremaux B., Gritzfeld J., Boa T., Yost C.K. (2009). Evaluation of host-specific *Bacteroidales* 16S rRNA gene markers as a complementary tool for detecting fecal pollution in a prairie watershed. Water Res..

[bib18] Gladstone B.P., Ramani S., Mukhopadhya I., Muliyil J., Sarkar R. (2011). Protective effect of natural rotavirus infection in an Indian birth cohort. New Engl. J. Med..

[bib19] Ishii S., Ksoll W.B., Hicks R.E., Sadowsky M.J. (2006). Presence and growth of naturalized *Escherichia coli* in temperate soils from Lake Superior watersheds. Appl. Environ. Microbiol..

[bib20] Jenkins M.W., Freeman M.C., Routray P. (2014). Measuring the safety of excreta disposal behavior in India with the new Safe San Index: reliability, validity and utility. Int. J. Environ. Res. Public Health.

[bib21] Jothikumar N., Kang G., Hill V.R. (2009). Broadly reactive TaqMan assay for real-time RT-PCR detection of rotavirus in clinical and environmental samples. J. Virol. Methods.

[bib22] Kildare B.J., Leutenegger C.M., McSwain B.S., Bambic D.G., Rajal V.B., Wuertz S. (2007). 16S rRNA-based assays for quantitative detection of universal, human-, cow-, and dog-specific fecal *Bacteroidales*: a Bayesian approach. Water Res..

[bib23] Kotloff K.L., Nataro J.P., Blackwelder W.C., Nasrin D., Farag T.H. (2013). Burden and aetiology of diarrhoeal disease in infants and young children in developing countries (the Global Enteric Multicenter Study, GEMS): a prospective, case-control study. Lancet.

[bib24] Kumar P., Jain M., Goel A.K., Bhadauria S., Sharma S.K., Kamboj D.V., Singh L., Ramamurthy T., Nair G.B. (2009). A large cholera outbreak due to a new cholera toxin variant of the *Vibrio cholerae* O1 El Tor biotype in Orissa, Eastern India. J. Med. Microbiol..

[bib25] Leclerc H., Mossel D.A., Edberg S.C., Struijk C.B. (2001). Advances in the bacteriology of the coliform group: their suitability as markers of microbial water safety. Annu. Rev. Microbiol..

[bib26] Levy K., Hubbard A.E., Nelson K.L., Eisenberg J.N. (2009). Drivers of water quality variability in northern coastal Ecuador. Environ. Sci. Technol..

[bib27] Lim S.S., Vos T., Flaxman A.D., Danaei G., Shibuya K. (2012). A comparative risk assessment of burden of disease and injury attributable to 67 risk factors and risk factor clusters in 21 regions, 1990–2010: a systematic analysis for the Global Burden of Disease Study 2010. Lancet.

[bib28] Liu L., Johnson H.L., Cousens S., Perin J., Scott S. (2012). Global, regional, and national causes of child mortality: an updated systematic analysis for 2010 with time trends since 2000. Lancet.

[bib29] Luby S.P., Agboatwalla M., Hoekstra R.M. (2011). The variability of childhood diarrhea in Karachi, Pakistan, 2002-2006. Am. J. Trop. Med. Hyg..

[bib30] Martella V., Banyai K., Matthijnssens J., Buonavoglia C., Ciarlet M. (2010). Zoonotic aspects of rotaviruses. Vet. Microbiol..

[bib31] Mattioli M.C., Pickering A.J., Gilsdorf R.J., Davis J., Boehm A.B. (2013). Hands and water as vectors of diarrheal pathogens in Bagamoyo, Tanzania. Environ. Sci. Technol..

[bib32] Odagiri M., Schriewer A., Hanley K., Wuertz S., Misra P.R., Panigrahi P., Jenkins M.W. (2015). Validation of *Bacteroidales* quantitative PCR assays targeting human and animal fecal contamination in the public and domestic domains in India. Sci. Total Environ..

[bib33] Pal B.B., Khuntia H.K., Samal S.K., Kar S.K., Patnaik B. (2010). Epidemics of severe cholera caused by El Tor *Vibrio cholerae* O1 Ogawa possessing the *ctxB* gene of the classical biotype in Orissa, India. Int. J. Infect. Dis..

[bib34] Patil S.R., Arnold B.F., Salvatore A.L., Briceno B., Ganguly S., Colford J.M., Gertler P.J. (2014). The effect of India's total sanitation campaign on defecation behaviors and child health in rural Madhya Pradesh: a cluster randomized controlled trial. PLoS Med..

[bib35] Pickering A.J., Boehm A.B., Mwanjali M., Davis J. (2010). Efficacy of waterless hand hygiene compared with handwashing with soap: a field study in Dar es Salaam, Tanzania. Am. J. Trop. Med. Hyg..

[bib36] Pickering A.J., Julian T.R., Mamuya S., Boehm A.B., Davis J. (2011). Bacterial hand contamination among Tanzanian mothers varies temporally and following household activities. Trop. Med. Int. Health.

[bib37] Pickering A.J., Julian T.R., Marks S.J., Mattioli M.C., Boehm A.B., Schwab K.J., Davis J. (2012). Fecal contamination and diarrheal pathogens on surfaces and in soils among Tanzanian households with and without improved sanitation. Environ. Sci. Technol..

[bib38] Power K.N., Nagy L.A. (1999). Relationship between bacterial regrowth and some physical and chemical parameters within Sydney's drinking water distribution system. Water Res..

[bib39] Rajal V.B., McSwain B.S., Thompson D.E., Leutenegger C.M., Wuertz S. (2007). Molecular quantitative analysis of human viruses in California stormwater. Water Res..

[bib40] Schmid-Hempel P., Frank S.A. (2007). Pathogenesis, virulence, and infective dose. PLoS Pathog..

[bib41] Schriewer A., Miller W.A., Byrne B.A., Miller M.A., Oates S. (2010). Presence of Bacteroidales as a predictor of pathogens in surface waters of the central California coast. Appl. Environ. Microbiol..

[bib42] Schriewer A., Odagiri M., Wuertz S., Misra P.R., Panigrahi P., Clasen T., Jenkins M.W. (2015). Human and animal fecal contamination of community water sources, stored drinking water and hands in rural India measured with validated microbial source tracking assays. Am. J. Trop. Med. Hyg..

[bib43] Synge B.A. (2000). Verocytotoxin-producing *Escherichia coli*: a veterinary view. Symp. Ser. Soc. Appl. Microbiol..

[bib44] Taniuchi M., Sobuz S.U., Begum S., Platts-Mills J.A., Liu J., Yang Z., Wang X.Q., Petri W.A., Haque R., Houpt E.R. (2013). Etiology of diarrhea in Bangladeshi infants in the first year of life analyzed using molecular methods. J. Infect. Dis..

[bib45] UNICEF (2013). Levels and Trends in Child Mortality.

[bib46] van der Wielen P.W.J.J., Medema G. (2010). Unsuitability of quantitative Bacteroidales 16S rRNA gene assays for discerning fecal contamination of drinking water. Appl. Environ. Microbiol..

[bib47] Verheyen J., Timmen-Wego M., Laudien R., Boussaad I., Sen S., Koc A., Uesbeck A., Mazou F., Pfister H. (2009). Detection of adenoviruses and rotaviruses in drinking water sources used in rural areas of Benin, West Africa. Appl. Environ. Microbiol..

[bib48] Vierheilig J., Farnleitner A.H., Kollanur D., Blöschl G., Reischer G.H. (2012). High abundance of genetic Bacteroidetes markers for total fecal pollution in pristine alpine soils suggests lack in specificity for feces. J. Microbiol. Methods.

[bib49] Young I., Smith B.A., Fazil A. (2015). A systematic review and meta-analysis of the effects of extreme weather events and other weather-related variables on Cryptosporidium and Giardia in fresh surface waters. J. Water Health.

