# Supplementary material for: Human fecal and pathogen exposure pathways in rural Indian villages and the effect of increased latrine coverage
Source: Water Res. 2016 Sep 1;100:232–44. doi: 10.1016/j.watres.2016.05.015 (PMC4907306; doi:10.1016/j.watres.2016.05.015)
Supplement: Supplementary file 1 [file mmc1.docx]

**Supplemental Material for:**

**Human fecal and pathogen exposure pathways in rural Indian villages and the effect of increased latrine coverage**

*Mitsunori Odagiri^a^, Alexander Schriewer^a^, Miles E. Daniels^b^, Stefan Wuertz^a,c^, Woutrina A. Smith^b^, Thomas Clasen^d,f^, Wolf-Peter Schmidt^f^, Yujie Jin^a^, Belen Torondel^f^, Pravas R. Misra^g^, Pinaki Panigrahi^e^, and Marion W. Jenkins ^a, f *^*

^a^ Department of Civil and Environmental Engineering, University of California, Davis, One Shields Avenue, Davis, California, USA

^b^ Department of Veterinary Medicine and Epidemiology, School of Veterinary Medicine, University of California, Davis, California, USA

^c^ Singapore Centre for Environmental Life Sciences Engineering (SCELSE), Nanyang Technological University, 60 Nanyang Drive, Singapore 637551

^d^ Department of Environmental Health, Rollins School of Public Health, Emory University, Atlanta, Georgia, USA

^e^ Departments of Epidemiology and Pediatrics, Center for Global Health and Development, College of Public Health, University of Nebraska Medical Center, Omaha, Nebraska, USA

^f^ Faculty of Infectious and Tropical Diseases, London School of Hygiene and Tropical Medicine, Keppel Street, London WC1E 7HT, UK

^g^ Asian Institute of Public Health, Bhubaneswar, Odisha, India

* Corresponding author, email: mwjenkins@ucdavis.edu

1. **Community water selection**

Two public tubewells (deep gourndwater), two private tubewells (shallow groundwater) and two open ponds (surface water) in each village were tested, unless fewer existed. Typically, study villages had 2-3 public tubewells and from 0 to 19 households at baseline with a private (shallow) tubewell. Thus, our set of sampled tubewells while not always exhaustive, including most and often all public tubewells (deeper groundwater drinking sources), and a random sample of private tubewells (shallow groundwater drinking sources), to represent local groundwater contamination in the village on the day of sampling, In the few cases where a village had more than two public tubewells or two public ponds, the two that were most heavily used (i.e. being used by more people) were identified through reconnaissance visits up to 5 days prior to sampling. Ponds samples were collected at the women’s access area used by young children who accompany their mothers. Sampled tubewells were those from which sampled households had drawn their drinking water, and when all sampled households used one public tubewell, another heavily used public tubewell was included, if it existed.

1. **Household stored drinking water (SDW) and hand rinse (HR) sample filtered volume**

Filtered volume varied depending on the sample type and turbidity. Mean filtered volume and standard deviation of SDW and HR samples were 456±137 mL and 288±55 mL, respectively.

1. **Thermotolerant coliform (TTC; also known as fecal coliform) measurement**

Sample volumes were filtered through a 0.45µm membrane (Millipore, Bedford, MA). Membranes were placed over lauryl sulfate media (Oxoid Limited, Basingstoke, Hampshire, UK) and incubated for 18 hours at 44°C. Detection limits ranged from 1-100 and 1-100,000 cfu/100 mL for improved and unimproved water sources, respectively, and from 1-20,000 cfu/100 mL and 30-71,200 cfu/two hands for SDW and HR samples, respectively. In the quantitative analysis of TTC, half the lower detection limit was assigned to non-detect (ND) samples (i.e. no colonies on a membrane), while 1.5 times the upper detection limit was assigned to too numerous to count (TNTC) samples (defined as more than 100 cfu per membrane).

1. **Molecular analysis**

*4.1. DNA/RNA extraction*

DNA and RNA were extracted simultaneously from RNALater-stabilized retentate samples (i.e. community water sources) and from molecular analysis filters (i.e. SDW and HR) using the PureLink Viral RNA/DNA Mini Kit (Life Technologies, Carlsbad, CA, USA), and a modified MoBio PowerWater RNA Isolation Kit (Mo Bio Laboratories Inc., Carlsbad, CA) ([Mattioli et al. 2012](#_ENREF_9)), respectively. For pathogenic *E. coli* virulent gene detection, DNA was extracted from 2013 FC analysis filters positive for colonies, using a modified MoBio PowerSoil DNA Isolation Kit (Mo Bio Laboratories Inc., Carlsbad, CA) according to Mattioli et al.([Mattioli et al. 2012](#_ENREF_9)).

*4.2. Sample limit of detection (SLOD) for each quantitative PCR assay*

SLOD varied depending on the processed sample volume. For HR, SLOD (gc per two hands) ranged from 308.1 - 4772.5, 116.3 - 1801.0 and 39.8 - 615.8 for total, human and animal-associated *Bacteroidales* markers, respectively. For SDW, SLOD (gc per mL) ranged from 0.4 - 4.9, 0.2 - 1.8 and 0.1 - 0.63 for total, human and animal-associated *Bacteroidales* markers, respectively. For community water sources, SLOD (gc per mL) ranged from 3.0 - 47.8, 1.1 - 11.1 and 0.4 - 4.5 for total, human and animal-associated *Bacteroidales* markers, respectively. SLOD (gc per mL) for rotavirus, adenovirus 40/41 and *V. cholerae* ranged from 0.03 – 0.5, 6.8 – 68.2 and 0.02 – 0.15, respectively.

1. **Correlations between TTC and fecal markers**

Analyses of households samples showed that concentrations of TTC were significantly correlated with those for animal (BacCow) and for total (BacUni) markers of *Bacteroidales* in SDW and in hand rinses, but were uncorrelated with human markers in all household sample types (see Table 3 in Schriewer et al. 2015). The correlations were strongest between TTC and BacCow markers. Here we examine correlation between TTC and MST markers in the public domain samples (i.e., community water sources) (see Table S4). Unlike in the domestic domain samples, significant correlation was not observed in any water source sample type. Although none reached significance, the highest observed correlation in pond and public TW samples was between TTC and animal (BacCow) markers, while in private TW samples, it was between TTC and total (BacUni) fecal markers (see Table S4).

1. **pH and Turbidity measurements**

We measured pH and turbidity of hand rinse samples collected in 2012 and all samples collected in 2013. Mean pH of mother and child HRs and SDW was 6.56±0.62, 6.46±0.60 and 7.09±0.59, respectively, while mean turbidity (NTU) of these sample types was 25.84±21.88, 33.96±27.82 and 8.44±7.05, respectively. For improved and unimproved water sources, mean pH of public and private tubewells and open ponds was 6.96±0.68, 6.72±0.62 and 6.80±0.48, respectively, while mean turbidity of these sample types was 11.91±17.91, 7.11±12.24 and 35.05±31.08, respectively.

Spearman rank correlation tests were used to explore correlation between physicochemical parameters (i.e. pH and turbidity) and MST and pathogen markers as described previously (Schriewer et al. 2015). In public and private tubewells and open pond samples, no significant correlations with pH or with turbidity were found. However, pH was positively correlated with total and with human fecal marker concentrations in SDW (Spearman ρ = 0.178, *P* < 0.01, and ρ = 0.232, *P* < 0.01, respectively) and with human fecal marker concentration in HRs (combined mother and child) (ρ = 0.143, *P* < 0.01). Turbidity also appeared to be associated with total, human and animal fecal marker concentrations in SDW (ρ = 0.301, *P* < 0.01; ρ = 0.240, *P* < 0.01, and ρ = 0.259, *P* < 0.01 respectively) and with total and animal fecal marker concentrations in HRs (combined) (ρ = 0.217, *P* < 0.01, and ρ = 0.158, *P* < 0.01, respectively).

We also performed t-test to compare differences in pH and turbidity between tubewell samples with and without pathogen positives (i.e. rotavirus, *Cryptosporidium* and *Giardia*), and did not find significant differences for any tested pathogens, although adenovirus and *V. cholerae* were not included in this analysis because none of 2013 samples were positive for them. This is likely due to low number of pathogen positives in 2013 samples (n = 1 to 4).

1. **Validation of pathogen assays**

*7.1 Confirmation against clinically positive stool samples from local diarrheal patients*

From May to July 2012, ninety fresh fecal samples were obtained from diarrheal patients in three local hospitals (Puri District Headquarter Hospital, Puri Government Area Hospital, and Capital Hospital in Bhubaneswar). Fecal samples were collected in sterile containers, placed on ice and transported to the Asian Institute of Public Health (AIPH) laboratory in Bhubaneswar. *Vibrio* spp and *E. coli* were detected using culture methods with thiosulfate citrate bile salts sucrose (TCBS) agar and MacConkey agar (Himedia), respectively. Among *Vibrio* spp. positive isolates, *Vibrio cholerae* were further identified based on the API 20E test (Bio-Merieux). For rotavirus and adenovirus detection, VIKIA Rota-Adeno test (Bio-Merieux) was used, following the manufacture protocol. DNA was then extracted from all *V. cholerae* isolates (n = 2) and all adenovirus positive fecal samples (n = 5), while RNA was extracted from ten rotavirus positive fecal samples (n = 10). We confirmed that *V. cholerae*, adenovirus and rotavirus qPCR assays used in this study amplified all tested extracts, indicating these assays could detect target pathogens locally prevalent.

For pathogenic *E. coli* PCR assay validation, ETEC and EPEC were obtained from the National Institute of Cholera and Enteric Diseases (NICED), Kolkota, and DNA was extracted while other strains were not available at the time of study. In addition, twenty *E. coli* isolates from the set of diarrheal patient fecal samples were also tested after DNA extraction. We confirmed that pathogenic *E. coli* PCR assays in this study amplified *lt1* (ETEC), *stlb* (ETEC), *aggR* (EAEC) and *eaeA* (EHEC/EPEC) using the locally collected diarrheal patient samples. However, we did not detect *stx1* (EHEC), *stx2* (EHEC) and *ipaH* (EIEC and *Shigella* spp.) in our tested fecal samples.

The diarrheal patient samples were also tested using the *Cryptosporidium* and *Giardia* assays and confirmed in >10% of samples. See Daniels et al. 2015 for details.

*7.2. qPCR product sequencing*

In order to validate qPCR pathogen assay results, qPCR amplicons positive for adenovirus (n=2, public ponds), *Vibrio cholera* (n=2, public tubwells) and rotavirus (n=10, public ponds) were sequenced. All results showed 98-100% similarity with target sequences of each pathogen, indicating that qPCR pathogens assay results were likely to be true positives.

1. **Statistical analysis**

*8.1. Effect of increased latrine coverage on transmission routes at village level*

We examined fecal and pathogen contamination rates at village-level for potential differences attributable to increased sanitation coverage. Specifically, we tested whether (1) the proportion of a village’s sampled households positive for a fecal marker, or (2) the proportion of a village’s tested improved sources (public and private tubewells) positive for any pathogen was lower in intervention than control villages. A household was considered positive when at least one of its three samples (i.e. SDW, mother’s HR, child’s HR) was detected with the target marker. Similarly, a tubewell was considered positive when at least one of five target pathogens (excluding pathogenic *E. coli*) was detected in the sample. Pathogenic *E. coli* was excluded because only villages sampled in 2013 were screened for pathogenic *E. coli*. Negative binomial regression was used to compare fractions positive in intervention and control villages. Dependent (outcome) variables were either the number of households positive for the fecal marker or the number of tubewells (i.e., improved groundwater sources) positive for any pathogen in the village on the day of sampling; the number of tested households/tubewells in the village was used as an offset variable.

*8.2. Microbial contamination of exposure pathways and child diarrhea*

To explore associations (1) between the level of human and of animal fecal contamination in MST sampled households and subsequent reported child diarrhea prevalence (at least one child in the sampled household with diarrhea as measured by the Sanitation Trial (Clasen et al. 2014)) and, (2) village-wide prevalence of pathogen contamination (detection) of community drinking and of domestic water sources and subsequent village-wide reported child diarrhea prevalence rates as measured by the Sanitation Trial (Ibid), a set of hypothesis was tested: higher levels of (more exposure pathways with) fecal contamination in households and more frequent detection of pathogens in improved and/or unimproved community water sources increased the risk of subsequent diarrhea in a child under 5 in a contaminated household, or in the case of community water sources, among all children in a village with contaminated community water sources. Study villages that had a Sanitation Trial diarrhea surveillance measurement within the 6 weeks following the date of environmental sampling (the date of detected contamination of an exposure pathway) were considered (n=43 MST study villages) based on the assumption that the incubation period for diarrhea pathogens in the body of an exposed child is no longer than 6 weeks. Of these 43 villages, 6 had their quarterly surveillance measurement collected within 7 days of environmental sampling and were excluded because the 7-day recall period included exposure conditions prior to the sampling date, leaving a total of 37 villages with a relevant diarrhea surveillance observation point. Details of diarrhea data collection procedures can be found elsewhere ([Clasen et al. 2014](#_ENREF_4)). The dependent (outcome) variables were (1) presence of at least one child reported with diarrhea over the 7-day recall period in the household (i.e. binary variable), and (2) the village-wide reported number of observed (all) under-5 children who experienced diarrhea in the 7-day recall period, with the total number of observed under-5 children used as the offset variable. For the first dependent variable, 14.9% (95%CI: 9.6-20.2) of 174 MST households (those in the 37 villages with a relevant (within-6-weeks) diarrhea surveillance observation point) had at least one child with diarrhea. Looking at the second dependent variable, mean of village-wide diarrhea prevalence (within 6 weeks) in the 37 villages was 11.6%±9.3%; the range of the number of observed under-5 children across the 37 MST villages was 24 to 51. To represent household-level contamination of tested fecal-oral exposure pathways, a categorical independent variable was used to indicate the level of human and of animal fecal contamination, defined as follows: (1) all pathways tested in the home (i.e. sample types: SDW, mother’s HR, and child’s HR) were negative for the target marker, (2) some but not all were positive, and (3) all were positive. To represent village-level rates of pathogen contamination of community drinking and domestic water sources, a continuous independent variable, the proportion of sampled community water sources detected with at least one tested pathogens (excluding pathogenic *E. coli* which was not measured for all study villages), was used. The association of diarrhea with pathogen contamination of community drinking water sources (deep and shallow groundwater tubewells) was examined separately from that of community ponds, which entails recreational, bathing and indirect pathways of exposures. Binary independent variables indicating the detection of a target pathogen in any tested community groundwater drinking amd in any tested domestic pond water source (any sampled tubewell, any sampled pond) in the village on the sampling date were also tested to shed light on possible pathogens of concern. Descriptive statistics of independent variables are summarized in Table S7 and S8.

Table S1. Detection methods for each target and samples tested for.

|  | Target | Method | Reference | Samples tested |
| --- | --- | --- | --- | --- |
| Microbial source tracking markers | Total *Bacteroidales* (BacUni) | qPCR | ([Kildare et al. 2007](#_ENREF_7)) | Public and private TW, open ponds, SDW and hand rinses |
|  | Human-associated Bacteroidales (BacHum) | qPCR | ([Kildare et al. 2007](#_ENREF_7)) |  |
|  | Hon-human-associated Bacteroidales (BacCow) | qPCR | ([Kildare et al. 2007](#_ENREF_7)) |  |
|  | Dog-associated Bacteroidales (BacCan) | qPCR | ([Kildare et al. 2007](#_ENREF_7)) |  |
|  |  |  |  |  |
| Conventional fecal indicator | Thermotolerant (or fecal) coliform | Culture | ([Eaton et al. 2012](#_ENREF_5)) | Public and private TW, open ponds, SDW and hand rinses |
|  |  |  |  |  |
| Viral pathogen | Rotavirus | RT-qPCR | ([Jothikumar et al. 2009](#_ENREF_6)) | Public and private TW, and open ponds |
|  | adenovirus 40/41 | qPCR | ([Rajal et al. 2007](#_ENREF_10)) |  |
|  |  |  |  |  |
| Bacterial pathogen | *Vibrio cholera* | qPCR | ([Blackstone et al. 2007](#_ENREF_1)) | Public and private TW, and open ponds |
|  | EAEC (*aggR*) and EPEC & EHEC (*eaeA*) | Multiplex PCR | ([Brandal et al. 2007](#_ENREF_2), [Ratchtrachenchai et al. 1997](#_ENREF_12), [Toma et al. 2003](#_ENREF_15), [Vidal et al. 2004](#_ENREF_16)) | Public and private TW, and open ponds  (fecal coliform positives in 2013) |
|  | EHEC (*stx1* & *stx2*) and EIEC & *Shigella* spp. (*ipaH*) | Multiplex PCR | ([Brian et al. 1992](#_ENREF_3), [Sethabutr et al. 1993](#_ENREF_13), [Toma et al. 2003](#_ENREF_15)) |  |
|  | ETEC (*lt1* & *st1b*) | Multiplex PCR | ([Brandal et al. 2007](#_ENREF_2), [Lopez-Saucedo et al. 2003](#_ENREF_8), [Rappelli et al. 2001](#_ENREF_11), [Stacy-Phipps et al. 1995](#_ENREF_14)) |  |
|  |  |  |  |  |
| Protozoan pathogen | *Cryptosporidium* | Fluorescent Microscopy |  | Public and private TW, and open ponds |
|  | *Giardia* | Fluorescent Microscopy |  |  |

Table S2. Detection frequencies, log _10_ mean of fecal markers in community water sources

| Sample type |  | No. of  samples^a^ | Detection frequency in percentage (95% CI) | | |  | Log Mean^b^ (gc/mL) | | |
| --- | --- | --- | --- | --- | --- | --- | --- | --- | --- |
|  |  |  | 2012 | 2013 | Combined |  | 2012 | 2013 | combined |
| Public TW | TTC (FC)^c^ | 105 | 48.8 | 15.6* | 28.6 (19.9-37.2) |  | 0.20 | -0.11 | 0.01 |
|  | Total^d^ | 110 | 39.5 | 56.7 | 50.0 (40.7-59.3) |  | 1.01 | 1.01 | 1.01 |
|  | Human^e^ | 109 | 4.7 | 1.5 | 2.8 (0.0-5.8) |  | -0.03 | -0.05 | -0.04 |
|  | Animal^f^ | 109 | 4.7 | 6.0 | 5.5 (1.2-9.8) |  | -0.48 | -0.46 | -0.47 |
|  |  |  |  |  |  |  |  |  |  |
| Private TW | TTC (FC) | 94 | 48.7 | 29.1 | 37.2 (27.5-47.0) |  | 0.49 | 0.07 | 0.24 |
|  | Total | 98 | 65.9 | 61.4 | 63.3 (53.7-72.8) |  | 1.41 | 1.17 | 1.27 |
|  | Human | 97 | 2.4 | 1.8 | 2.1 (0.0-4.9) |  | -0.05 | -0.04 | -0.04 |
|  | Animal | 96 | 12.2 | 3.6 | 7.3 (2.1-12.5) |  | -0.28 | -0.35 | -0.32 |
|  |  |  |  |  |  |  |  |  |  |
| Improved water sources (public and private TW) | TTC (FC) | 199 | 48.8 | 21.8 | 32.7 (26.1-39.2) |  | 0.34 | -0.02 | 0.12 |
|  | Total | 208 | 52.4 | 58.9 | 56.3 (49.5-63.0) |  | 1.20 | 1.09 | 1.13 |
|  | Human | 206 | 3.6 | 1.6 | 2.4 (0.3-4.5) |  | -0.04 | -0.05 | -0.05 |
|  | Animal | 205 | 8.3 | 5.0 | 6.3 (3.0-9.7) |  | -0.38 | -0.41 | -0.40 |
|  |  |  |  |  |  |  |  |  |  |
| Open ponds | TTC (FC) | 90 | 91.2 | 87.5 | 88.9 (82.4-95.4) |  | 3.50 | 3.46 | 3.48 |
|  | Total | 93 | 100.0 | 100.0 | 100.0 |  | 4.25 | 3.97 | 4.07 |
|  | Human | 90 | 8.6 | 3.6 | 5.6 (0.8-10.3) |  | 0.55 | 0.52 | 0.53 |
|  | Animal | 92 | 77.1 | 54.4* | 63.0 (53.2-72.9) |  | 1.34 | 0.94 | 1.09 |

^a^ Number of samples was different among assays because we eliminated samples having Sample Limit of Detection (SLOD) outliers.

^b^ A half SLOD was assigned for samples below detection limits

^c^ Thermotolerant coliform (TTC), also known as Fecal coliform

^d^ Total fecal marker (BacUni)

^e^ Human fecal marker (BacHum)

^f^ Animal fecal marker (BacCow)

* denotes that difference of detection frequencies between 2012 and 2013 was statistically significant (χ^2^, *p* < 0.05)

Table S3. Detection frequencies and log_10_ mean of fecal markers in stored drinking water (SDW) and hand rinses

| Sample type |  | No. of  samples^a^ | Detection frequency in percentage  (95% CI) | | |  | Log Mean^b^  (gc/mL) or (gc/two hands) | | |
| --- | --- | --- | --- | --- | --- | --- | --- | --- | --- |
|  |  |  | 2012 | 2013 | Combined |  | 2012 | 2013 | combined |
| SDW | TTC (FC)^c^ | 330 | 81.9 | 68.0* | 73.6 (68.9-78.4) |  | 2.42 | 2.06 | 2.20 |
|  | Total^d^ | 346 | 91.7 | 28.0* | 52.9 (47.6-58.1) |  | 1.78 | 0.25 | 0.84 |
|  | Human^e^ | 345 | 18.5 | 19.8 | 19.4 (15.2-23.6) |  | -0.57 | -0.35 | -0.43 |
|  | Animal^f^ | 346 | 50.0 | 21.8* | 32.7 (27.7-37.6) |  | -0.25 | -0.77 | -0.57 |
|  |  |  |  |  |  |  |  |  |  |
| Hand  Rinses | TTC (FC) | 657 | 65.0 | 54.9* | 58.9 (55.1-62.7) |  | 2.55 | 2.32 | 2.41 |
|  | Total | 685 | 98.1 | 51.4* | 69.9 (66.5-73.4) |  | 5.39 | 3.84 | 4.45 |
|  | Human | 682 | 27.4 | 12.1* | 18.0 (15.1-20.9) |  | 2.50 | 2.20 | 2.32 |
|  | Animal | 684 | 89.8 | 59.4* | 71.5 (68.1-74.9) |  | 3.89 | 2.91 | 3.29 |

^a^ Number of samples was different among assays because we eliminated samples having Sample Limit of Detection (SLOD) outliers.

^b^ A half SLOD was assigned for samples below detection limits

^c^ Thermotolerant (or Fecal) coliform

^d^ Total fecal marker (BacUni)

^e^ Human fecal marker (BacHum)

^f^ Animal fecal marker (BacCow)

* denotes that difference of detection frequencies between 2012 and 2013 was statistically significant (χ^2^, *p* < 0.05)

Table S4. Public domain correlations between thermotolerant coliform (TTC) and *Bacteroidales* associated fecal markers in ponds, public and private tubewells (TWs) in 2012.

|  |  | No. of samples | Spearman correlation | |
| --- | --- | --- | --- | --- |
|  |  |  | ρ | *P* value |
| Pond TTC | Pond BacUni | 34 | 0.14 | 0.429 |
|  | Pond BacHum | 34 | 0.021 | 0.904 |
|  | Pond BacCow | 34 | 0.179 | 0.310 |
| Public TW TTC | Public TW BacUni | 41 | 0.038 | 0.814 |
|  | Public TW BacHum | 41 | -0.206 | 0.196 |
|  | Public TW BacCow | 41 | 0.105 | 0.512 |
| Private TW TTC | Private TW BacUni | 39 | 0.285 | 0.079 |
|  | Private TW BacHum | 39 | - |  |
|  | Private TW BacCow | 39 | 0.264 | 0.105 |
| Improved drinking water (public and private TWs) TTC | Improved BacUni | 80 | 0.17 | 0.133 |
|  | Improved BacHum | 80 | -0.145 | 0.198 |
|  | Improved BacCow | 80 | 0.197 | 0.079 |

Table S5. Detection frequencies, log _10_ median and log _10_ mean of pathogens in community water sources

| Sample type |  | No. of  samples^a^ |  | Detection frequency in percentage (95% CI) | | | | Log Mean^b^ (gc/mL) or (cyst or oocyst /20L)^c^ | | | | |
| --- | --- | --- | --- | --- | --- | --- | --- | --- | --- | --- | --- | --- |
|  |  |  |  | | 2012 | 2013 | combined |  | 2012 | 2013 | combined |  |
| Public TW | rotavirus | 109 |  | | 18.6 | 1.5* | 8.3 (3.1-13.4) |  | -1.03 | -1.56 | -1.36 |  |
|  | adenovirus | 109 |  | | 7.0 | 0.0* | 2.8 (0.0-5.8) |  | 0.75 | 0.71 | 0.73 |  |
|  | *V. cholerae* | 109 |  | | 32.6 | 0.0* | 12.8 (6.6-19.1) |  | -1.17 | -1.94 | -1.64 |  |
|  | Any pathogenic  *E.coli* | 63 |  | | - | 1.6 | 1.6 (0.0-4.7) |  | - | - | -^d^ |  |
|  | *Cryptosporidium* | 111 |  | | 25.6 | 5.9* | 13.5 (7.2-19.9) |  | 1.47 | 0.98 | 1.34 |  |
|  | *Giardia* | 111 |  | | 27.9 | 1.5* | 11.7 (5.7-17.7) |  | 1.49 | 0.95 | 1.45 |  |
|  |  |  |  | |  |  |  |  |  |  |  |  |
| Private TW | rotavirus | 98 |  | | 12.2 | 5.3 | 8.2 (2.7-13.6) |  | -1.19 | -1.44 | -1.34 |  |
|  | adenovirus | 98 |  | | 4.9 | 0.0 | 2.1 (0.0-4.9) |  | 0.77 | 0.73 | 0.75 |  |
|  | *V. cholerae* | 98 |  | | 65.9 | 0.0* | 27.6 (18.7-36.4) |  | -0.34 | -1.92 | -1.26 |  |
|  | Any pathogenic  *E.coli* | 54 |  | | - | 0.0 | 0.0 |  | - | - | -^d^ |  |
|  | *Cryptosporidium* | 98 |  | | 12.2 | 3.5 | 7.1 (2.0-12.2) |  | 1.38 | 1.51 | 1.42 |  |
|  | *Giardia* | 98 |  | | 36.6 | 5.3* | 18.4 (10.7-26.0) |  | 1.44 | 1.47 | 1.45 |  |
|  |  |  |  | |  |  |  |  |  |  |  |  |
| Improved  water  sources (public and private TW) | rotavirus | 207 |  | | 15.5 | 3.3* | 8.2 (4.5-12.0) |  | -1.11 | -1.51 | -1.35 |  |
|  | adenovirus | 206 |  | | 6.0 | 0.0* | 2.4 (0.3-4.5) |  | 0.76 | 0.72 | 0.73 |  |
|  | *V. cholerae* | 207 |  | | 48.8 | 0.0* | 19.8 (14.4-25.2) |  | -0.76 | -1.93 | -1.46 |  |
|  | Any pathogenic  *E.coli* | 117 |  | | - | 0.9 | 0.9 (0.0-2.5) |  | - | - | -^d^ |  |
|  | *Cryptosporidium* | 209 |  | | 19.0 | 4.8* | 10.5 (6.4-14.7) |  | 1.44 | 1.46 | 1.36 |  |
|  | *Giardia* | 209 |  | | 32.1 | 3.2* | 14.8 (10.0-19.7) |  | 1.16 | 1.34 | 1.45 |  |
|  |  |  |  | |  |  |  |  |  |  |  |  |
| Open ponds | rotavirus | 90 |  | | 22.9 | 58.2* | 44.4 (34.2-54.7) |  | -0.27 | 0.79 | 0.39 |  |
|  | adenovirus | 90 |  | | 2.9 | 1.8 | 2.2 (0.0-5.3) |  | 1.27 | 1.29 | 1.28 |  |
|  | *V. cholerae* | 90 |  | | 0.0 | 0.0 | 0.0 |  | -1.45 | -1.39 | -1.41 |  |
|  | Any pathogenic  *E.coli* | 54 |  | | - | 48.1 | 48.1 (34.8-61.5) |  | - | - | -^d^ |  |
|  | *Cryptosporidium* | 94 |  | | 57.1 | 25.4* | 37.2 (27.5-47.0) |  | 1.90 | 1.91 | 1.90 |  |
|  | *Giardia* | 94 |  | | 82.9 | 69.5 | 74.5 (65.7-83.3) |  | 2.31 | 2.01 | 2.14 |  |

^a^ Number of samples was different among assays because we eliminated samples having Sample Limit of Detection (SLOD) outliers.

^b^ A half SLOD was assigned for samples below detection limits

^c^ For *Cryptosporidium* and *Giardia*, units are cyst per 20L and oocysts per 20L, respectively.

^d^ Pthogenic *E.coli* was detected using conventional PCR. Hence, no quantitative data is available.

* Denotes that difference of detection frequencies between 2012 and 2013 was statistically significant (χ^2^, *p* < 0.05)

Table S6. Detection frequencies of pathogenic *E.coli* virulent genes in community water sources collected in 2013

| Sample type | No. of  samples |  | Detection frequency in percentage (95% CI) | | | | | | | |
| --- | --- | --- | --- | --- | --- | --- | --- | --- | --- | --- |
|  |  |  | Any pathogenic *E.coli* virulent genes | *aggR* (EAEC) | *eaeA* (EHEC/EPEC) | *stx1* (EHEC) | *stx2* (EHEC) | *ipaH* (EIEC and *Shgella* spp.) | *lt1* (ETEC) | *st1b* (ETEC) |
| Public TW | 63 |  | 1.6 | 0.0 | 0.0 | 0.0 | 0.0 | 0.0 | 1.6 | 0.0 |
| Private TW | 54 |  | 0.0 | 0.0 | 0.0 | 0.0 | 0.0 | 0.0 | 0.0 | 0.0 |
| Improved water sources * | 117 |  | 0.9  (0.0-2.5) | 0.0 | 0.0 | 0.0 | 0.0 | 0.0 | 0.9  (0.0-2.5) | 0.0 |
| Open Ponds | 54 |  | 48.1  (34.8-61.5) | 18.5  (8.3-28.9) | 24.1  (12.7-35.5) | 3.7  (0-8.7) | 9.3  (1.5-17.0) | 1.9  (0-5.4) | 5.6  (0-11.7) | 11.1  (2.7-19.5) |

* Public and private TWs combined.

Table S7. Proportion of sampled community water sources detected with at least one tested pathogen (except pathogenic *E. coli*)

| Variables | Number of villages | Mean | Standard deviation |
| --- | --- | --- | --- |
| Proportion of improved drinking water sources (i.e. tested tubewells) in a village positive for any of five tested pathogens | 37^a^ | 0.32 | 0.36 |
|  |  |  |  |
| Proportion of surface water sources (i.e. tested pubic ponds) in the village positive for any of five tested pathogens | 36^b^ | 0.89 | 0.30 |

^a^ Of 60 study villages, 37 had a Sanitation Trial diarrhea surveillance 7-day recall period prevalence observation visit between 1 and 6 weeks of the date of environmental sampling (see Supplemental Material section 8.2).

^b^ One of the 37 villages had no community ponds.

Table S8. Frequency of villages where any tested tubewell or any tested pond source was positive for the target pathogen.

| Variables | Number of villages | Detection frequency (%) | 95% CI | |
| --- | --- | --- | --- | --- |
| Pathogen detected in any tested tubewell water source in a village: |  |  |  |  |
| Rotavirus (Y/N) | 37^a^ | 27.0 | 12.7 | 41.3 |
| Adenovirus (Y/N) | 37 | 8.1 | 0.0 | 16.9 |
| *V. cholerae* (Y/N) | 37 | 29.7 | 15.0 | 44.5 |
| *Cryptosporidium* (Y/N) | 37 | 29.7 | 15.0 | 44.5 |
| *Giardia* (Y/N) | 37 | 32.4 | 17.3 | 47.5 |
| Any pathogenic *E. coli* (Y/N) | 22^b^ | 2.7 | 0.0 | 13.2 |
|  |  |  |  |  |
| Pathogen detected in any tested pond water source in a village: |  |  |  |  |
| Rotavirus (Y/N) | 36^c^ | 61.1 | 45.2 | 77.0 |
| Adenovirus (Y/N) | 36 | 2.8 | 0.0 | 8.1 |
| *V. cholera* (Y/N) | 36 | Not detected |  |  |
| *Cryptosporidium* (Y/N) | 36 | 44.4 | 28.2 | 60.7 |
| *Giardia* (Y/N) | 36 | 83.3 | 71.2 | 95.5 |
| Any pathogenic *E. coli* (Y/N) | 22* | 59.1 | 38.5 | 79.6 |

^a^ Of 60 study villages, 37 had a Sanitation Trial diarrhea surveillance 7-day recall period prevalence observation visit between 1 and 6 weeks of the date of environmental sampling (see Supplemental Material section 8.2).

^b^ Only study villages sampled in 2013 had their community water sources tested for pathogenic E. coli virulence genes.

^c^ One of the 37 villages had no community ponds.

Figure S1. Temporal variability in the proportion of sampled improved drinking water sources (public and private tubewells) positive for total fecal markers in each village. Black dot and empty circle denote year of 2012 and 2013, respectively. Each dot is one village.

Figure S2. Forest plots of relative risks of viral and protozoan pathogens in community water sources in Sanitation Trial intervention over control villages, stratified by year (i.e. (a) 2012 and (b) 2013). Pathogens whose detection rates were too small to calculate relative risks remained blank in the figure.

Figure S3. Forest plots of relative risks of detecting fecal markers in community water sources in Sanitation Trial intervention over control villages, stratified by year (i.e. (a) 2012 and (b) 2013). Fecal markers whose detection rates were too small to calculate relative risks remained blank in the figure.

**References**

Blackstone, G.M., Nordstrom, J.L., Bowen, M.D., Meyer, R.F., Imbro, P. and DePaola, A. (2007) Use of a real time PCR assay for detection of the ctxA gene of Vibrio cholerae in an environmental survey of Mobile Bay. J Microbiol Methods 68(2), 254-259.

Brandal, L.T., Lindstedt, B.A., Aas, L., Stavnes, T.L., Lassen, J. and Kapperud, G. (2007) Octaplex PCR and fluorescence-based capillary electrophoresis for identification of human diarrheagenic Escherichia coli and Shigella spp. J Microbiol Methods 68(2), 331-341.

Brian, M.J., Frosolono, M., Murray, B.E., Miranda, A., Lopez, E.L., Gomez, H.F. and Cleary, T.G. (1992) Polymerase chain reaction for diagnosis of enterohemorrhagic Escherichia coli infection and hemolytic-uremic syndrome. J Clin Microbiol 30(7), 1801-1806.

Clasen, T., Boisson, S., Routray, P., Torondel, B., Bell, M., Cumming, O., Ensink, J., Freeman, M., Jenkins, M., Odagiri, M., Ray, S., Sinha, A., Suar, M. and Schmidt, W.-P. (2014) Effectiveness of a rural sanitation programme on diarrhoea, soil-transmitted helminth infection, and child malnutrition in Odisha, India: a cluster-randomised trial. The Lancet Global Health 2(11), e645-e653.

Daniels, M., Smith, W.A., Shrivastava, A., Sahu, P., Odagiri, M., Misra, P.R., Panigrahi, P., Suar, M., Clasen, T. and Jenkins, M.W. (2015) Cryptosporidium and Giardia in Humans, Domestic Animals, and Village Water Sources in Coastal Odisha, India. Am J Trop Med Hyg 93(3), 596-600.

Eaton, D.A., Clesceri, S.L., Rice, W.E., Greenberg, E.A. and Franson, H.A.M. (2012) Standard Methods for the Examination of Water and Wastewater, American Public Health Association, American Water Works Association, Water Environment Federation, Washington D.C.

Jothikumar, N., Kang, G. and Hill, V.R. (2009) Broadly reactive TaqMan assay for real-time RT-PCR detection of rotavirus in clinical and environmental samples. J Virol Methods 155(2), 126-131.

Kildare, B.J., Leutenegger, C.M., McSwain, B.S., Bambic, D.G., Rajal, V.B. and Wuertz, S. (2007) 16S rRNA-based assays for quantitative detection of universal, human-, cow-, and dog-specific fecal Bacteroidales: a Bayesian approach. Water Res 41(16), 3701-3715.

Lopez-Saucedo, C., Cerna, J.F., Villegas-Sepulveda, N., Thompson, R., Velazquez, F.R., Torres, J., Tarr, P.I. and Estrada-Garcia, T. (2003) Single multiplex polymerase chain reaction to detect diverse loci associated with diarrheagenic Escherichia coli. Emerging Infectious Diseases 9(1), 127-131.

Mattioli, M.C., Pickering, A.J., Gilsdorf, R.J., Davis, J. and Boehm, A.B. (2012) Hands and Water as Vectors of Diarrheal Pathogens in Bagamoyo, Tanzania. Environ Sci Technol.

Rajal, V.B., McSwain, B.S., Thompson, D.E., Leutenegger, C.M. and Wuertz, S. (2007) Molecular quantitative analysis of human viruses in California stormwater. Water Res 41(19), 4287-4298.

Rappelli, P., Maddau, G., Mannu, F., Colombo, M.M., Fiori, P.L. and Cappuccinelli, P. (2001) Development of a set of multiplex PCR assays for the simultaneous identification of enterotoxigenic, enteropathogenic, enterohemorrhagic and enteroinvasive Escherichia coli. New Microbiol 24(1), 77-83.

Ratchtrachenchai, O.A., Subpasu, S. and Ito, K. (1997) Investigation on enteroaggregative Escherichia coli infection by multiplex PCR. Bulletin of the Department of Medical Sciences 39, 211-220.

Sethabutr, O., Venkatesan, M., Murphy, G.S., Eampokalap, B., Hoge, C.W. and Echeverria, P. (1993) Detection of Shigellae and enteroinvasive Escherichia coli by amplification of the invasion plasmid antigen H DNA sequence in patients with dysentery. J Infect Dis 167(2), 458-461.

Schriewer, A., Odagiri, M., Wuertz, S., Misra, P.R., Panigrahi, P., Clasen, T. and Jenkins, M.W. (2015) Human and animal fecal contamination of community water sources, stored drinking water and hands in rural India measured with validated microbial source tracking assays. Am J Trop Med Hyg 93(3), 509-516.

Stacy-Phipps, S., Mecca, J.J. and Weiss, J.B. (1995) Multiplex PCR assay and simple preparation method for stool specimens detect enterotoxigenic Escherichia coli DNA during course of infection. J Clin Microbiol 33(5), 1054-1059.

Toma, C., Lu, Y., Higa, N., Nakasone, N., Chinen, I., Baschkier, A., Rivas, M. and Iwanaga, M. (2003) Multiplex PCR assay for identification of human diarrheagenic Escherichia coli. J Clin Microbiol 41(6), 2669-2671.

Vidal, R., Vidal, M., Lagos, R., Levine, M. and Prado, V. (2004) Multiplex PCR for diagnosis of enteric infections associated with diarrheagenic Escherichia coli. J Clin Microbiol 42(4), 1787-1789.
